# Timing the Landmark Events in the Evolution of Clear Cell Renal Cell Cancer: TRACERx Renal

**DOI:** 10.1016/j.cell.2018.02.020

**Published:** 2018-04-19

**Authors:** Thomas J. Mitchell, Samra Turajlic, Andrew Rowan, David Nicol, James H.R. Farmery, Tim O’Brien, Inigo Martincorena, Patrick Tarpey, Nicos Angelopoulos, Lucy R. Yates, Adam P. Butler, Keiran Raine, Grant D. Stewart, Ben Challacombe, Archana Fernando, Jose I. Lopez, Steve Hazell, Ashish Chandra, Simon Chowdhury, Sarah Rudman, Aspasia Soultati, Gordon Stamp, Nicos Fotiadis, Lisa Pickering, Lewis Au, Lavinia Spain, Joanna Lynch, Mark Stares, Jon Teague, Francesco Maura, David C. Wedge, Stuart Horswell, Tim Chambers, Kevin Litchfield, Hang Xu, Aengus Stewart, Reza Elaidi, Stéphane Oudard, Nicholas McGranahan, Istvan Csabai, Martin Gore, P. Andrew Futreal, James Larkin, Andy G. Lynch, Zoltan Szallasi, Charles Swanton, Peter J. Campbell, the TRACERx Renal Consortium

**Affiliations:** 1 Cancer Genome Project, Wellcome Trust Sanger Institute, Hinxton CB10 1SA, UK; 2 Academic Urology Group, Department of Surgery, Addenbrooke’s Hospitals NHS Foundation Trust, University of Cambridge, Hills Road, Cambridge CB2 0QQ, UK; 3 Translational Cancer Therapeutics Laboratory, the Francis Crick Institute, 1 Midland Rd, London NW1 1AT, UK; 4 Renal and Skin Units, The Royal Marsden National Health Service (NHS) Foundation Trust, London SW3 6JJ, UK; 5 CRUK Cambridge Institute, University of Cambridge, Robinson Way, Cambridge CB2 0RE, UK; 6 Guy’s and St Thomas’ National Health Service (NHS) Foundation Trust, Great Maze Pond, London SE1 9RT, UK; 7 Department of Pathology, Cruces University Hospital, Biocruces Institute, University of the Basque Country (UPV/EHU), Barakaldo, Spain; 8 Experimental Histopathology Laboratory, The Francis Crick Institute, 1 Midland Road, London NW1 1AT, UK; 9 Interventional Radiology Department, The Royal Marsden National Health Service (NHS) Foundation Trust, London SW3 6JJ, UK; 10 Big Data Institute, University of Oxford, Old Road Campus, Oxford OX3 7FZ, UK; 11 Bioinformatics and Biostatistics STP, Francis Crick Institute, 1 Midland Road, London NW1 1AT, UK; 12 Hôpital Européen Georges Pompidou 20, rue Leblanc, 75908 Paris, France; 13 Cancer Research UK Lung Cancer Centre of Excellence, University College London Cancer Institute, Paul O’Gorman Building, 72 Huntley Street, London WC1E 6BT, UK; 14 Department of Physics of Complex Systems, Eotvos Lorand University, Budapest, Hungary; 15 The University of Texas MD Anderson Cancer Center, Department of Genomic Medicine, Houston, TX 77030, USA; 16 School of Medicine, University of St. Andrews, North Haugh, St. Andrews KY16 9TF, UK; 17 Centre for Biological Sequence Analysis, Technical University of Denmark, Lyngby, Denmark; 18 Children’s Hospital Informatics Program at the Harvard-MIT Division of Health Sciences and Technology (CHIP@HST), Harvard Medical School, Boston, MA, USA; 19 Department of Medical Oncology, University College London Hospitals, 235 Euston Rd, Fitzrovia, London NW1 2BU, UK; 20 Department of Haematology, University of Cambridge, Cambridge CB2 2XY, UK

**Keywords:** clear cell renal cell carcinoma, cancer evolution, chromothripsis

## Abstract

Clear cell renal cell carcinoma (ccRCC) is characterized by near-universal loss of the short arm of chromosome 3, deleting several tumor suppressor genes. We analyzed whole genomes from 95 biopsies across 33 patients with clear cell renal cell carcinoma. We find hotspots of point mutations in the 5′ UTR of *TERT*, targeting a MYC-MAX-MAD1 repressor associated with telomere lengthening. The most common structural abnormality generates simultaneous 3p loss and 5q gain (36% patients), typically through chromothripsis. This event occurs in childhood or adolescence, generally as the initiating event that precedes emergence of the tumor’s most recent common ancestor by years to decades. Similar genomic changes drive inherited ccRCC. Modeling differences in age incidence between inherited and sporadic cancers suggests that the number of cells with 3p loss capable of initiating sporadic tumors is no more than a few hundred. Early development of ccRCC follows well-defined evolutionary trajectories, offering opportunity for early intervention.

## Highlights

• Novel hotspot of driver mutations in 5′-UTR repressor of *TERT*, expanding telomeres
• Most common cause of 3p loss is a chromothripsis event, generating concurrent 5q gain
• t(3;5) event occurs in childhood or adolescence, decades before tumor diagnosed
• Initial clonal expansion after 3p loss starts from only a few hundred cells

## Graphical Abstract

**Figure:**
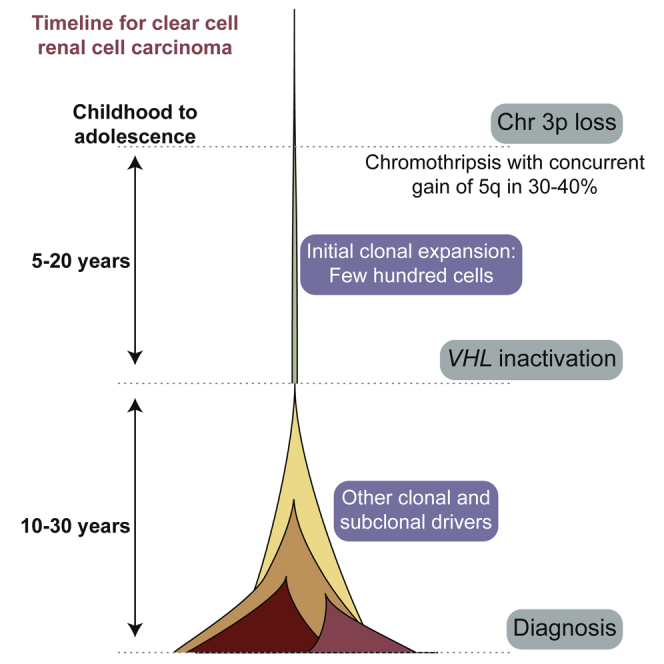

## Teaser

Combination of whole-genome sequencing analysis and a multi-region sampling approach provides insights into the nature and timing of key oncogenic events in clear cell renal cell carcinoma, depicts the evolutionary trajectories of tumors in patients and highlights the opportunity for early intervention.

## Introduction

Cancers of the kidney develop in an estimated 300,000 people worldwide every year, with approximately half dying from the disease (Fitzmaurice et al., 2015). The commonest histological subtype is clear cell renal cell carcinoma (ccRCC), a tumor believed to arise from the epithelial cells of the proximal convoluted tubule of the nephron (Frew and Moch, 2015).

The genome of clear cell renal cell carcinoma is distinctive. Loss of the short arm of chromosome 3 is the critical genetic event, found in >90% patients (Beroukhim et al., 2010, Shen et al., 2011, Cancer Genome Atlas Research Network, 2013, Zbar et al., 1987). The deleted region always encompasses four tumor suppressor genes that are frequent targets for inactivating point mutations on the other chromosomal copy: *VHL* (point mutations in 60%–70% patients; epigenetic silencing in a further 5%–10%), *PBRM1* (40%), *BAP1* (10%), and *SETD2* (10%) (Dalgliesh et al., 2010, Sato et al., 2013, Cancer Genome Atlas Research Network, 2013, Varela et al., 2011). The second most frequent genetic event in clear cell renal cell carcinoma is gain of chromosome 5q, seen in 65%–70% of patients (Beroukhim et al., 2010, Shen et al., 2011, Cancer Genome Atlas Research Network, 2013), with *SQSTM1* one of the likely target genes (Li et al., 2013).

Recent exome sequencing studies have highlighted the considerable intra-tumoral heterogeneity of clear cell renal cell carcinomas (Gerlinger et al., 2012, Gerlinger et al., 2014). In growing to sizes of several centimeters in diameter, these tumors often comprise several geographically localized subclones. Interestingly, chromosome 3p loss and, when present, *VHL* point mutations are always on the trunk of the phylogenetic tree, suggesting that they are key early events in cancer development.

Studies of somatic mutations in clear cell renal cell carcinoma to date have primarily focused on protein-coding genes. As a result, the mechanism of chromosome 3p loss has not been well characterized, nor the role of non-coding driver mutations. Here, using a multi-region sampling approach, we report whole genome sequences from 95 clear cell renal cell carcinoma biopsies across 33 patients.

## Results

### Whole-Genome Sequencing of Clear Cell Renal Cell Carcinomas

TRACERx Renal is a prospective cohort study of patients with RCC, which aims to assess the evolutionary trajectories of clear cell renal cell carcinoma (Turajlic and Swanton, 2017). In particular, multi-region sampling of the primary cancer and any metastases is used to generate high-resolution information on the timing of driver mutations, level of intratumoral heterogeneity, and presence of parallel evolution in each patient. To date, 100 patients in TRACERx Renal have been profiled with exome and targeted gene sequencing and these data are presented in the companion papers to this one (Turajlic et al., 2018a, Turajlic et al., 2018b).

We performed whole genome sequencing to an average 67x depth on 128 kidney biopsies, together with matched germline DNA, from 36 patients. The tumor cell fraction was not sufficient in 33 biopsies (including 17 biopsies from normal adjacent kidney) to accurately call somatic aberrations—the dataset analyzed here therefore represents whole genomes of 95 cancer biopsies from 33 patients (Table S1). Clinically, the patients had the typical age range, stage, and size of tumors for sporadic clear cell renal cell carcinoma (Table S2). We used our validated bioinformatics pipelines to identify somatic substitutions, indels, copy number alterations, and structural variants (Campbell et al., 2008, Jones et al., 2016, Raine et al., 2015, Raine et al., 2016).

**Figure 1:**
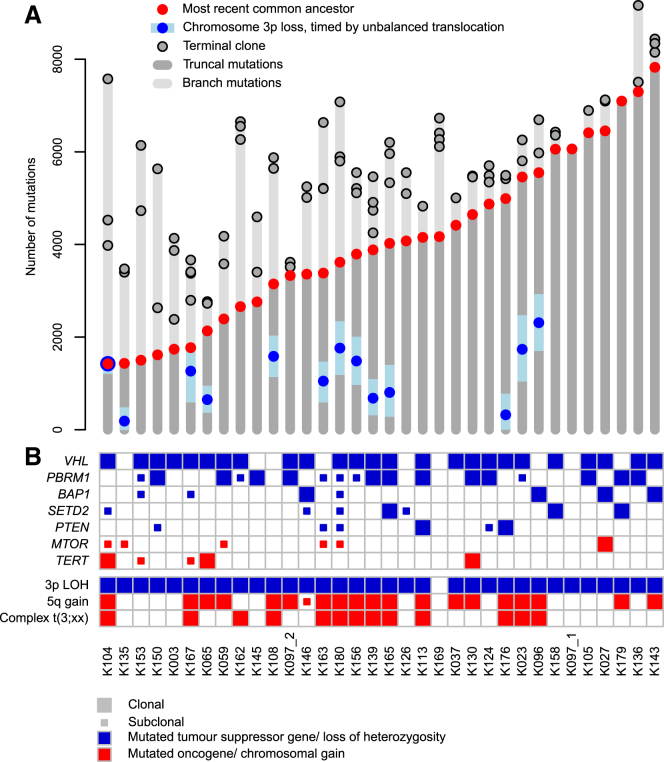
The Clonality of Driver Events and the Relative Timing of 3p Loss in Clear Cell Renal Cell Carcinoma (A) Mutation burden for 34 independent tumors derived from 33 patients. For each tumor, the number of mutations present in the most recent common ancestor and each of the terminal subclones are annotated. The estimated mutational time at which chromosome 3p is lost with 95% CIs has been annotated for those tumors harboring unbalanced translocations with 3p. One patient (K097) developed two independent tumors denoted K097_1 and K097_2. (B) Presence and clonality of driver mutations and copy number aberrations. Driver mutations include those previously reported and that are present in at least 3 independent tumors from this cohort. For cases where a clonal mutation in the WGS data has been detected as subclonal in the more spatially detailed panel data (Turajlic et al., 2018a, Turajlic et al., 2018b), the mutation has been amended in this figure as subclonal. See also Tables S1 and S2.

We identified an average of 7,680 unique somatic substitutions and 1,193 indels per patient, but with a 3-fold variation in numbers across patients (Figure 1A; Table S2). The landscape of coding driver mutations and recurrent copy number alterations was typical for clear cell renal cell carcinoma (Figure 1B). There was a high level of concordance between driver mutation calls made in whole genome and targeted panel sequencing (STAR Methods).

### Non-coding Driver Mutations in the 5′ UTR of *TERT*

Whether there are driver mutations in non-coding regions of the genome has not been extensively explored in clear cell renal cell carcinoma. We assessed these using a model of the background mutation rate across the genome that combines the observed mutation spectrum with genome-wide covariates known to affect mutation rate (Martincorena et al., 2017, Nik-Zainal et al., 2016) (Table S3).

**Figure 2:**
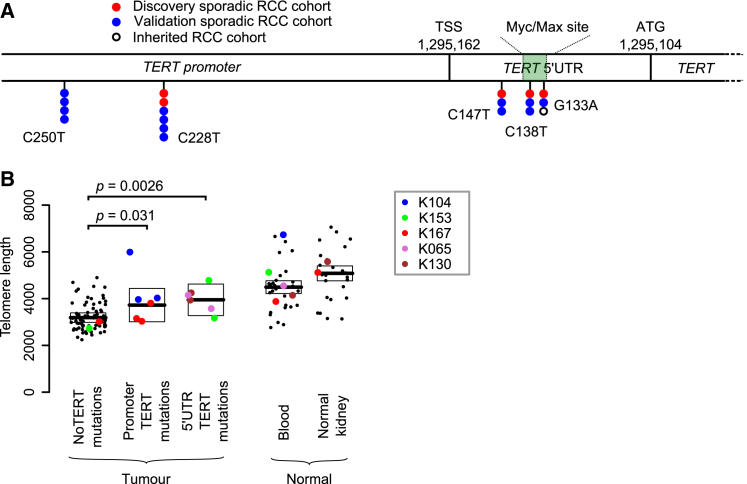
Recurrent Canonical and 5′ UTR *TERT* Mutations Increase Telomere Length (A) The genomic location of the canonical promoter and 5′ UTR mutations in this discovery cohort, a validation cohort (Table S5) and an inherited clear cell renal cell carcinoma cohort. (B) Estimated telomere lengths for all samples sequenced. The colored points correspond to samples that contained *TERT* mutations in some or all of the biopsies. The boxes indicate median and interquartile range. See also Tables S3 and S4.

Only one non-coding region had a statistically significant excess of mutations: the 5′ UTR and promoter of the telomerase reverse transcriptase gene, *TERT* (q = 0.016). This region harbored somatic mutations in 5 patients from our cohort of 33 (15%) (Figure 2A), of which two were subclonal. Interestingly, the mutation sites observed in our clear cell renal cell carcinoma data included three positions in the 5′ UTR of *TERT*, located 15, 24, and 29 base pairs downstream of the transcription start site (Figure 2A). These are different positions from the canonical promoter hotspots mutated in *TERT* across a wide range of cancers, especially melanomas (Horn et al., 2013, Huang et al., 2013), although we did see mutations at these sites as well (Figure 2A). In chromophobe renal cancer, structural variants activating *TERT* are common (Davis et al., 2014), but we detected neither genomic rearrangements nor copy number aberrations near *TERT* in this cohort of clear cell renal cell carcinomas.

To assess whether the 5′ UTR mutations were recurrent, we screened the promoter and 5′ UTR of *TERT* in an additional 377 samples from 94 patients with clear cell renal cell carcinoma by capillary sequencing (Table S4). This identified 13 patients with non-coding *TERT* mutations (13.5% of the cohort). The mutations were present clonally in 10 patients and subclonally in the other 3 and were distributed across the two canonical promoter sites and the three hotspots in the 5′ UTR identified in the discovery screen (Figure 2A). In our combined dataset, we find no association between *TERT* status and tumor grade or metastatic spread (p = 0.6 and p = 0.4, respectively), nor was there an association with chromothripsis events.

The three mutated loci in the *TERT* 5′ UTR fall in or very near to an E-box sequence (CACGTG), a motif known to bind the MYC-MAX-MAD1 family of proteins (Sabò and Amati, 2014). This specific E-box element was first shown to bind MYC in B lymphocytes, leading to transcriptional activation (Wu et al., 1999). However, the effects of this element on transcriptional activity are variable across cell types (Kyo et al., 2000), explained in part by competition for the binding site between MYC, which upregulates expression, and MAD1, which acts as a repressor (Oh et al., 2000). In renal cancer cells, this element acts mainly as a repressor, a function that is abrogated by mutation of the binding site (Horikawa et al., 2002). The implication is that the mutations we observe diminish binding of a repressor, probably MAD1, to the E-box, leading to loss of the usual transcriptional suppression of *TERT* in kidney cells.

Unfortunately, we do not have expression data to compare *TERT* expression in samples with and without 5′ UTR mutations, but we could directly estimate telomere lengths from the genome sequencing data (Farmery et al., 2017) (STAR Methods). If the mutations act to abolish the active repression of *TERT* transcription, then samples carrying these mutations should have longer telomeres. We used linear mixed models adjusted for age to determine the difference between groups. As previously reported (Barthel et al., 2017), tumors have shorter telomere lengths than normal tissue (p = 2.2 × 10^−16^), presumably reflecting the greater replicative drive and consequent telomere attrition in cancer cells. As predicted, samples with the canonical *TERT* promoter mutations and indeed those with 5′ UTR mutations did, on average, have longer telomeres than wild-type samples (p = 0.031 and p = 0.0026, respectively) (Figure 2B, Table S5). Thus, 5′ UTR hotspot mutations presumably act through lengthening telomeres to promote replicative immortality.

### Simultaneous Chromosome 3p Loss and 5q Gain through Chromothripsis

Despite being the most frequent genetic abnormality in clear cell renal cell carcinoma, the mechanisms underlying chromosome 3p loss have not been systematically characterized. Cytogenetic analyses have shown that unbalanced translocations between chromosomes 3 and 5 occur in 6%–60% of primary clear cell renal cell carcinoma samples (Klatte et al., 2009, Kovacs et al., 1987, Pavlovich et al., 2003) and renal cancer cell lines (Ali et al., 2013, Yang et al., 2000). We used paired-end sequencing data to reconstruct the genomic rearrangements causing 3p loss. Of the 33 tumors, we could pinpoint the position on chromosome 3p at which heterozygosity was lost in 30 cases—in 29 of these, we could identify the actual structural change driving loss of heterozygosity.

**Figure 3:**
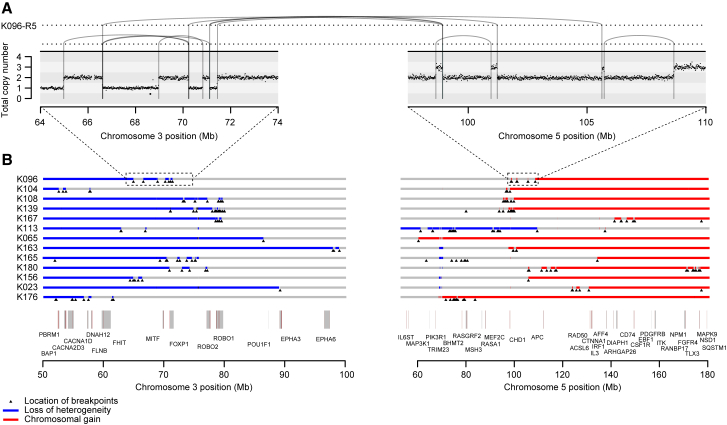
Recurrent Complex Unbalanced Translocations between Chromosomes 3 and 5 (A) Intra and inter-chromosomal re-arrangements and their effect on the copy number profile from an indicative tumor sample. All tumor samples containing these events are shown in Figure S1. (B) The genomic location of all breakpoints from all tumors that harbored translocations between chromosomes 3 and 5. Regions that had undergone loss of heterozygosity are shown in blue; those that have undergone gains are shown in red. See also Figure S2 and Table S6.

**Figure 4:**
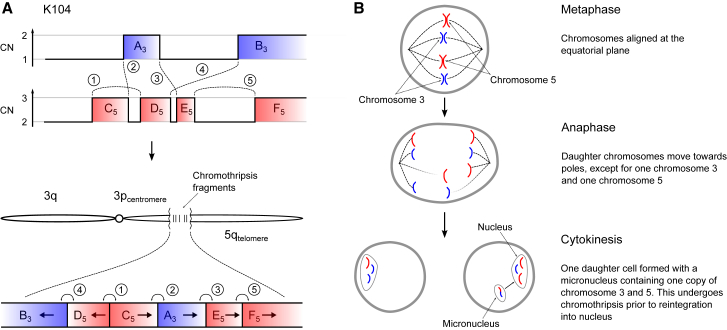
Schematic Illustrating How Chromothripsis Generates a Derivative t(3;5) Chromosome (A) In one of the simpler clusters of rearrangements observed, breakpoints, and copy number (CN) aberrations on chromosomes 3 and 5 allow unequivocal reconstruction of the orientation and localization of regions retained after chromothripsis. The derivative chromosome contains chromosome 3q, a centromeric region of 3p, the chromothripsis fragments, and the telomeric portion of chromosome 5q. (B) Schematic showing one possible mechanism whereby chromothripsis may result in the unbalanced translocation between chromosomes 3 and 5.

**Figure S1:**
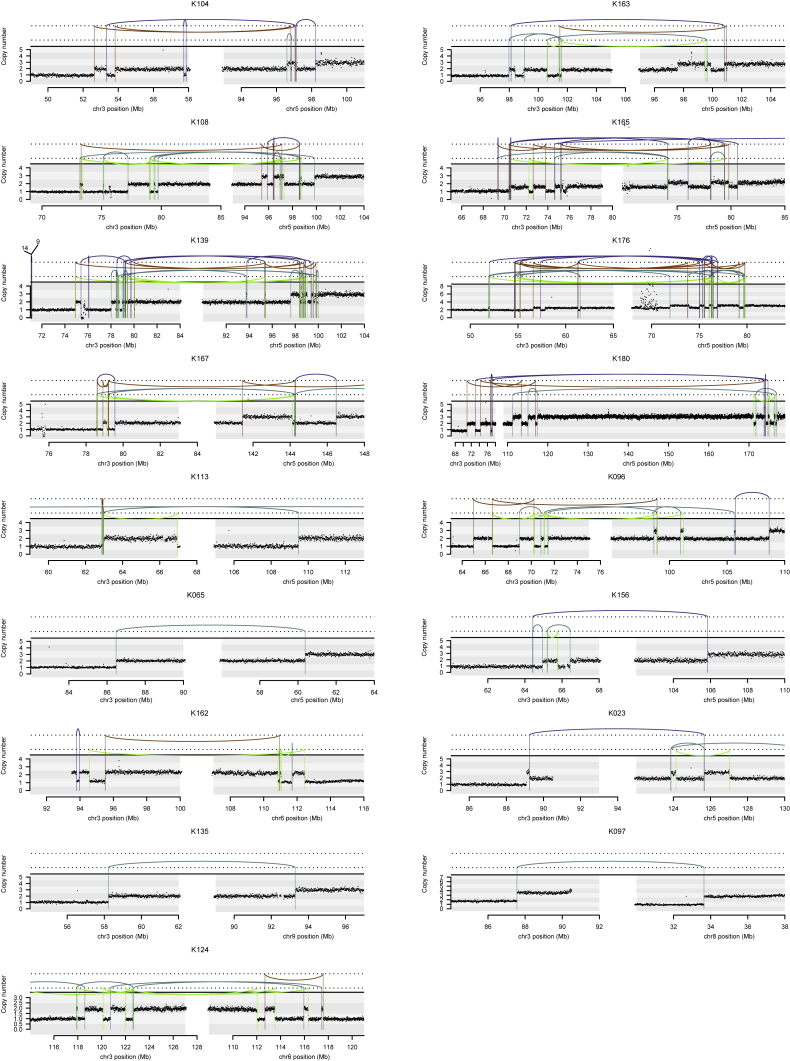
Intra and Inter-chromosomal Rearrangements Affecting Chromosome 3, Related to Figure 3 Copy number is plotted as number of reads in a given genomic window, corrected for ploidy of the tumor and aberrant cell fraction. Somatic structural variants are shown as arcs joining the two sides of the breakpoint, colored by orientation of the two segments. Blue lines, deletion orientation; brown lines, tandem duplication orientation; blue-green lines, head-to-head inverted orientation; bright green lines, tail-to-tail inverted orientation.

The most frequent pattern of chromosome 3p loss in the cohort, affecting 13 (43%) of the 30 tumors with known 3p LOH breakpoints, was rearrangement between 3p and 5q. In all but one of these tumors, the overall consequence was to lose one copy of chromosome 3p and gain an extra copy of chromosome 5q in the same event. In only two of these patients was the event a straightforward unbalanced translocation. In the remainder, there were groups of 5–30 rearrangements focally clustered on chromosomes 3p and 5q (Figures 3 and S1). These had the hallmarks of chromothripsis, a catastrophic mutational process in which one or a few chromosomes suffer multiple breaks simultaneously, with the resulting fragments being joined in random order (Stephens et al., 2011). In particular, the oscillating copy number profiles, clustered rearrangements, random orientation of breakpoint ends and phasing of rearrangements to one haplotype (Figure 4A) are all distinguishing genomic features of chromothripsis (Korbel and Campbell, 2013).

The explanation that best fits the copy number and rearrangement data is that chromothripsis results in a single t(3;5) derivative chromosome, alongside one wild-type chromosome 3 and two copies of wild-type chromosome 5 (Figure 4B). In our samples, the t(3;5) derivative chromosome consists of, in order: the intact long arm of 3q; the chromosome 3 centromere; a small portion of 3p from near the centromere; shuffled genomic fragments of 3p and 5q arising from chromothripsis; and the telomeric portion of 5q. Other sequences of events are formally possible, but implausible for several reasons (“Inference of chromothripsis” in the STAR Methods).

**Figure S2:**
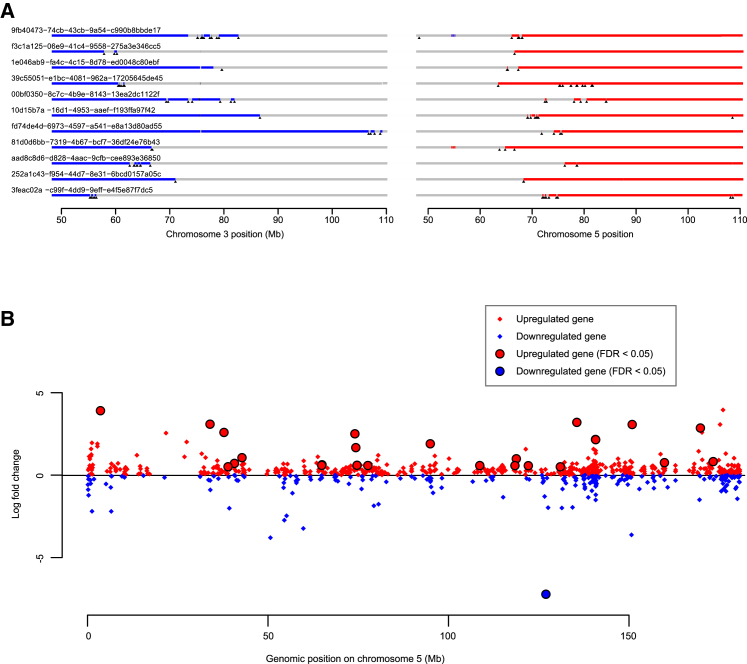
Analysis of TCGA Data, Related to Figure 3 (A) The genomic location of all breakpoints from all tumors that harbored translocations between chromosomes 3 and 5. Regions with loss of heterozygosity are shown in blue; those with copy number gain in red. Positions of breakpoints are marked with black triangles. (B) Fold-change in expression of all genes on chromosome 5 for those tumors that had a gain of 5q compared to those with wild-type chromosome 5. Significantly differentially expressed genes (FDR < 0.05) are highlighted.

To assess whether the t(3;5) chromothripsis events were recurrent in other cohorts, we re-examined whole genome sequencing data from the TCGA clear cell renal cell carcinoma study (The Cancer Genome Atlas Research Network, 2013). This revealed a similar overall frequency of events generating simultaneous loss of chromosome 3p and gain of 5q, seen in 11 tumors out of 40 studied (28%) (Figure S2A). In particular, clustered and interlocking rearrangements on chromosomes 3p and 5q confirm that chromothripsis is the predominant mechanism causing this critical driving event.

Although t(3;5) events were the commonest pattern causing chromosome 3p loss in our cohort (13/30 patients), they were by no means the only mechanism. We observed a range of other, less frequent structural abnormalities driving 3p loss. Two patients had chromothripsis events involving 3p and 6q, which led to losses on both chromosomes (Figure S1), and a further six patients had unbalanced translocations with various chromosomes other than 5q. Three patients had loss of the whole of chromosome 3 and three had loss of the entire short arm. Only two patients had simple interstitial deletions on chromosome 3p. In one patient, we were unable to map the event causing 3p loss.

### Chromothripsis on 3p and 5q Acts through Copy Number Change

We were surprised that a complex event such as chromothripsis was the major process causing the copy number changes on chromosomes 3p and 5q, rather than say simple unbalanced translocation. We examined the location of breakpoints to ascertain whether the clusters of rearrangements had generated a particular genomic configuration that might be recurrent across patients (Figures 3B and S2A). In fact, across patients, there was no obvious common region of chromothripsis on either 3p or 5q beyond the requirement to lose all four tumor suppressor genes on 3p and duplicate the terminal portion of 5q.

This suggests that the reason chromothripsis is so frequent is mechanistic. Our hypothesis is that any event that gains a chromosome arm must occur after S-phase and the most efficient way to couple this with simultaneous loss of another chromosome arm is through mitotic catastrophe. Indeed, *in vitro* studies show that mitotic errors induced by either microtubule dysfunction, causing lagging chromosomes (Zhang et al., 2015), or telomere crisis, causing anaphase bridges (Maciejowski et al., 2015), can result in similar copy number alterations and clusters of rearrangements between two chromosomes.

The key genes for the copy number gain on chromosome 5q remain mysterious, with several, including *SQSTM1*, proposed as targets (Li et al., 2013, Cancer Genome Atlas Research Network, 2013). From the TCGA cohort, we identified genes with differential expression in patients with 5q gains versus those with baseline copy number (Figure S2B; Data S1). Many genes in the duplicated regions of 5q are indeed upregulated (Table S6), consistent with the proposal that large-scale aneuploidy acts through a net tilt in the balance between dosage of growth-promoting and growth-suppressing genes (Davoli et al., 2013).

### Burden of Somatic Substitutions Correlates Linearly with Age

**Figure 5:**
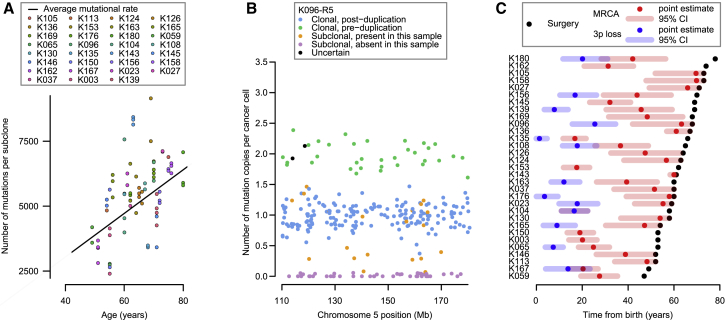
Mutational Burden and the Chronological Loss of Chromosome 3p (A) Mutational burden of subclones compared to age at surgery (points), annotated with the patient-specific and cohort average mutational rate (black line). (B) The estimated number of copies per cancer cell of each mutation in the duplicated region of 5q for an indicative sample. Mutations may be assigned as clonal and pre-duplication (green) or post-duplication (blue), subclonal and present (orange) or absent (purple) in this sample, or uncertain (black). (C) Estimated age of 3p loss (blue points), the most common recent ancestor (red) and surgical excision (black) with 95% CIs (shaded bars). See also Figures S3, S4, and S5 and Data S1.

To assess whether point mutations in clear cell renal cell carcinoma occur at constant rate, we correlated the age of diagnosis with the burden of base substitutions in each subclone across the cohort using mixed effects models (Figure 5A; Data S1; STAR Methods). Three key observations emerge. First, there is a statistically significant and linear correlation of mutation burden with age in this cohort, estimated at 87 mutations/year (95% confidence interval [CI]: 80–94; p < 0.001). Second, there is variation among patients in the rate at which mutations accumulate, with the between-patient standard deviation in mutation rate estimated at 17 mutations/year. Third, within a given patient’s tumor, different subclones have broadly similar mutation burdens (Figure 5A), suggesting that each subclone has been accumulating mutations at the same steady rate since clonal divergence.

**Figure S3:**
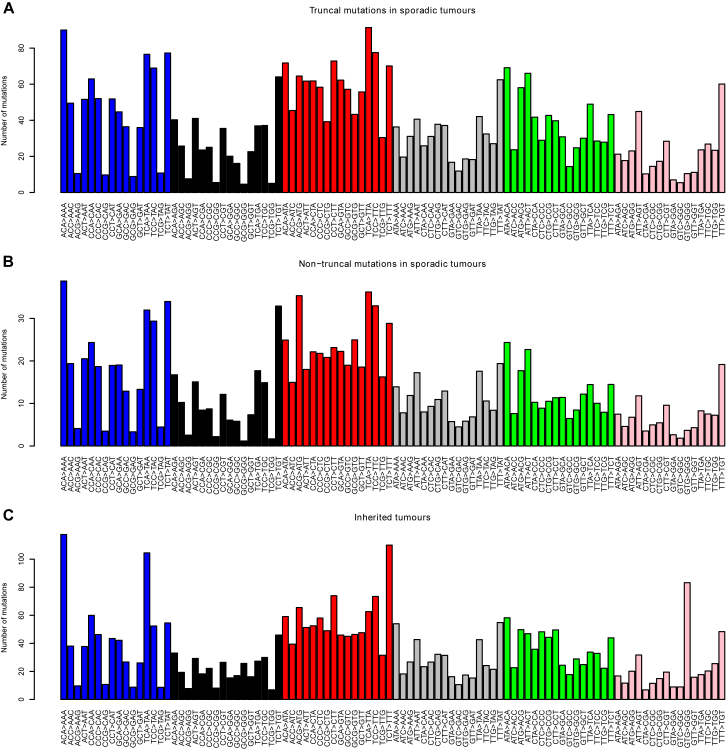
The Average Number of Mutations by Mutational Context, Related to Figure 5 (A) Truncal mutations in sporadic tumors. (B) Non-truncal mutations in sporadic tumors. (C) Mutations in inherited ccRCCs in von Hippel-Lindau disease. Bars represent average number of mutations per tumor of the six different types (C > A, C > G, C > T, T > A, T > C, T > G) with each of the 16 different combinations of base before and after the mutated base.

Taken together, these data suggest that somatic mutations in kidney cells accumulate at a constant rate throughout life. Further evidence for this comes from the mutational spectrum observed in clear cell kidney cancers both in this cohort (Figure S3) and in others (Alexandrov et al., 2013). The vast majority of mutations appear to arise from two mutational processes (so-called signatures 1 and 5) that are universal across cancer types and show linear correlation with age in both cancer (Alexandrov et al., 2015) and normal tissue (Blokzijl et al., 2016).

### Timing the Landmark Events of Clear Cell Renal Cell Carcinoma Development

We can estimate when large duplications occurred from the proportion of point mutations in that region that were duplicated. Essentially, any mutation that was on 5q before the t(3;5) event occurred will be duplicated along with the whole chromosome arm (and hence present on two of the three copies of 5q); any mutation that occurs subsequently will be present on only one of the three copies of 5q. From the fraction of mutations present on two versus one copy of 5q, and measures of the mutation rate, we can estimate the chronological age at which the duplication occurred. This approach has been used in several previous studies (Durinck et al., 2011, Nik-Zainal et al., 2012), and the methodology has been formally developed (Greenman et al., 2012).

**Figure S4:**
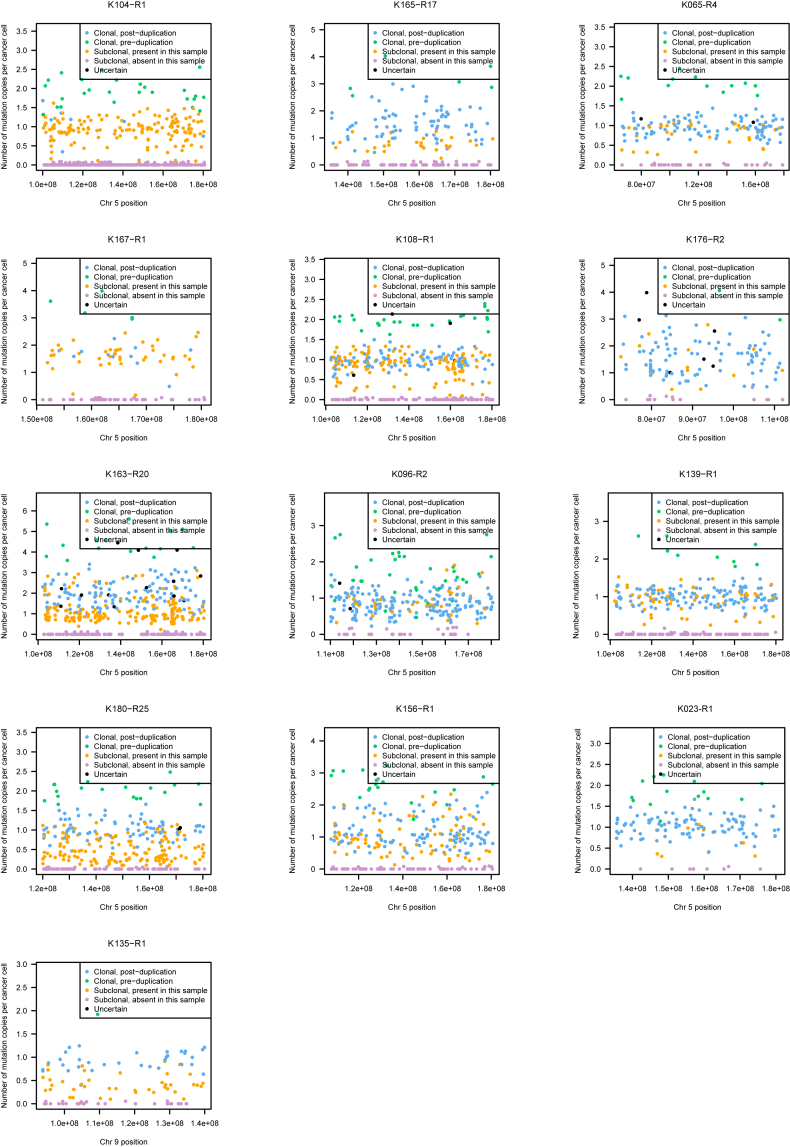
Number of Copies of Each Mutation per Cancer Cell for Regions of Chromosome 5q Gain, Related to Figure 5 All patients with 5q gain in the setting of t(3;5) unbalanced translocations are shown. The estimated number of copies per cancer cell of each mutation in the duplicated region of 5q is plotted. Mutations may be assigned as clonal and pre-duplication (green) or post-duplication (blue); subclonal and present (orange) or absent (purple) in this sample; or uncertain (black).

We estimated the age at which the t(3;5) translocation events occurred from mutations on the duplicated region of chromosome 5q (Figures 5B and S4; Data S1). Mutations can be divided into four categories: those present on two copies of 5q (green points, Figure 5B), clonal mutations present on one copy of 5q (blue points), mutations that are subclonal in the cancer as a whole and are found in the given sample (orange points), and subclonal mutations absent from the given sample (purple points). To estimate the ages of patients when t(3;5) events occurred, we used the patient-specific estimates of mutation rate generated by the linear mixed effects models (Figure 5A), with correction for the clonal structure and type of copy number gain (Greenman et al., 2012, Nik-Zainal et al., 2012) (STAR Methods). We used bootstrapping to generate 95% CIs for this estimate, incorporating the uncertainty in both the numbers of pre-duplication mutations and estimates of the patient-specific mutation rate.

In most patients, only a small fraction of mutations on the duplicated region of 5q were present on two chromosomal copies (Figures 5B and S4). This implies that the 5q duplication occurs surprisingly early in life. Formal statistical analysis estimated that t(3;5) events occurred during childhood or adolescence for the majority of patients in our cohort, 30–50 years before the kidney cancer was diagnosed (Figure 5C).

**Figure S5:**
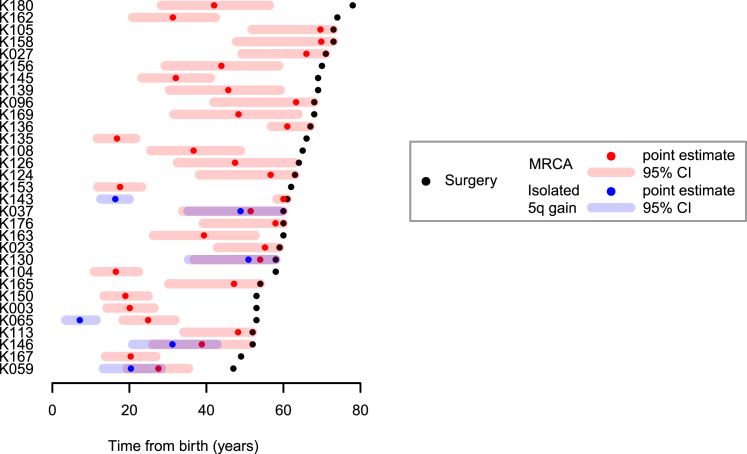
Age at which Isolated Chromosome 5 Gains Occurred, Related to Figure 5 Shown are the estimated ages at which patients acquired a clonal 5q gain (blue), not occurring with 3p loss, relative to the age of diagnosis (black) and estimated age at which the most recent common ancestor (MRCA) emerged (red). Shading indicates 95% confidence intervals for the estimated age.

One patient (K135) had a t(3;9) unbalanced translocation with loss of 3p and gain of 9q, which we also estimated to have occurred early in childhood (Figure 5C). In contrast, several patients had gains of 5q that were not linked with 3p and these appeared to have occurred at a much wider range of ages than the t(3;5) events (Figure S5). These data suggest that the key driver for the early timing of t(3;5) events is chromosome 3p loss.

These estimates depend on the assumption of a constant mutation rate throughout life. We explored other relationships between mutational burden and age, such as including a quadratic term for age, effectively allowing for the mutation rate to increase with age. Not only did this model fail to significantly improve the correlation between age and mutational burden, the estimated age of 3p loss increased by less than a year. Similarly, if we allowed for different periods of time for the clonal expansion between the occurrence of the last detectable mutation and tumor diagnosis, the estimated age of t(3;5) events did not increase.

In addition to timing the t(3;5) events, we can also estimate the age at which the most recent common ancestor of the tumor emerged. This cell is defined as the ancestral cell from which all current tumor cells derived, and its arrival demarcates the clonal mutations, found in all tumor cells, from the subclonal mutations, found in a fraction of tumor cells. In this cohort, we estimate a wide range of ages at which the most recent common ancestor emerged, from early adulthood through to late middle age (Figure 5C). This is reminiscent of previous exome data in which the relative length of the trunk of the phylogenetic tree across kidney cancer patients was strikingly variable (Gerlinger et al., 2014).

In one patient (K104), the estimated age of the t(3;5) chromothripsis was virtually the same as the estimated age at which the most recent common ancestor emerged (Figure 5C). This suggests that in this patient, the t(3;5) event was what triggered the last complete selective sweep in the tumor—the most recent common ancestor was likely the cell that underwent the chromothripsis catastrophe. If so, the clonal *VHL* and *TERT* driver mutations also seen in this tumor must have preceded the chromothripsis. For all the other patients in whom we could time the 3p loss and 5q gain, however, there was a delay of years to decades between the 3p loss and the emergence of the most recent common ancestor. This implies that the typical sequence of events is for 3p loss to be the initiating driver event, often occurring through t(3;5) chromothripsis. This is followed by one or more other driver mutations—these trigger the clonal expansion of the most recent common ancestor.

### Similar Landscape of Clear Cell Renal Cell Carcinoma in von Hippel-Lindau Disease

Germline mutations in *VHL* result in a syndrome known as von Hippel-Lindau disease, characterized by a high penetrance of clear cell renal cell carcinomas, together with hemangioblastomas of the retina, brain, and spine, and a handful of other tumor types (Nielsen et al., 2016). Renal cancers in von Hippel-Lindau disease begin to emerge in young adulthood, with a cumulative incidence of 70%–80% by the age of 60 years (Ong et al., 2007). It is known that the wild-type allele of *VHL* is universally deleted in these cancers, as expected for a classic two-hit tumor suppressor gene (Maher et al., 1990).

**Figure 6:**
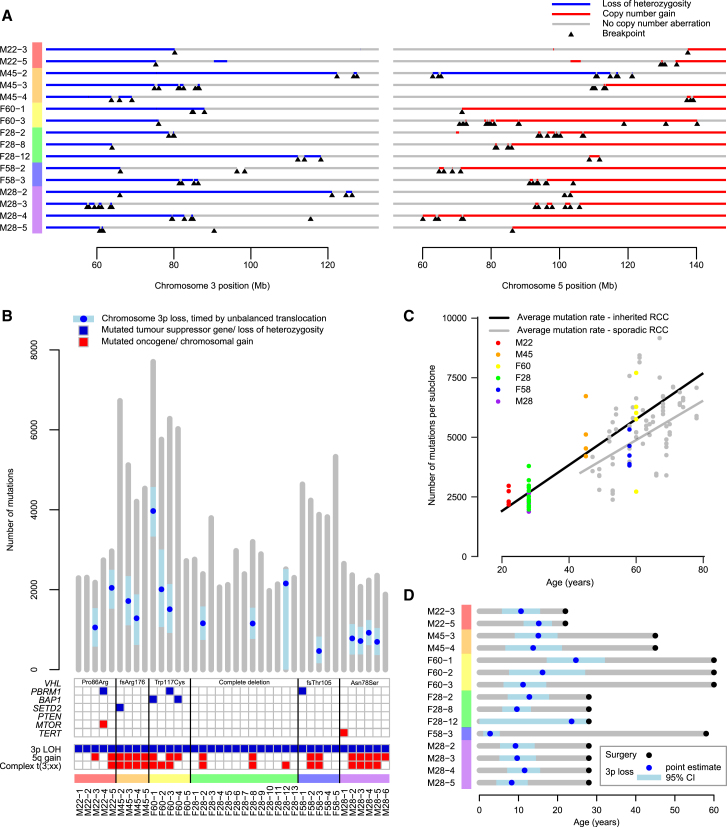
Similar Genomic Landscape of Inherited Clear Cell Renal Cell Carcinoma (A) Breakpoints and copy number aberrations for samples with von Hippel-Lindau disease that had translocations between 3p and 5q. (B) Driver events and molecular timing of 3p loss with 95% CIs. (C) Mutational burden versus age. (D) Estimated age of 3p loss and surgical excision with 95% CIs. See also Figures S6 and S7.

**Figure S6:**
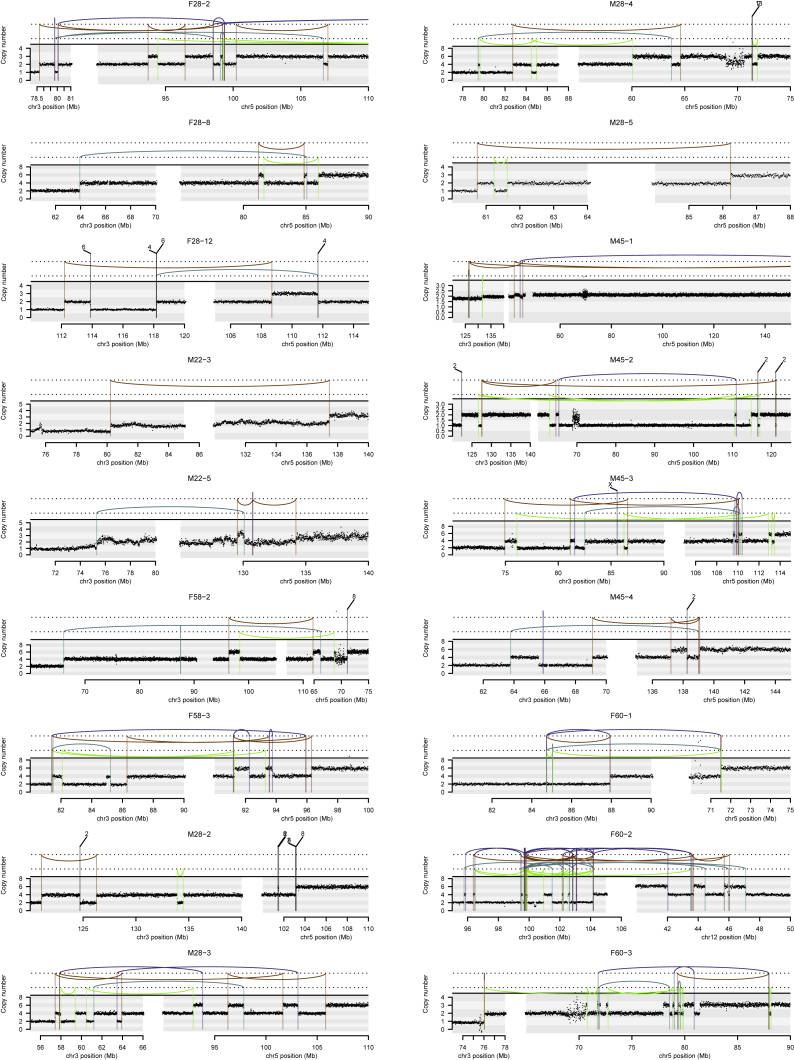
Intra- and Inter-chromosomal Rearrangements Affecting Chromosome 3 in the Inherited von Hippel-Lindau Disease Dataset, Related to Figure 6 Copy number is plotted as number of reads in a given genomic window, corrected for ploidy of the tumor and aberrant cell fraction. Somatic structural variants are shown as arcs joining the two sides of the breakpoint, colored by orientation of the two segments. Blue lines, deletion orientation; brown lines, tandem duplication orientation; blue-green lines, head-to-head inverted orientation; bright green lines, tail-to-tail inverted orientation.

Recently, whole genomes have been sequenced for 40 clear cell renal cell carcinomas from 6 patients with von Hippel-Lindau disease (Fei et al., 2016). To compare inherited with sporadic cases, we reanalyzed these data using our pipelines to establish how the wild-type *VHL* allele was lost in these cancers. As seen in the sporadic cases, we find clustered rearrangements between chromosomes 3p and 5q, reminiscent of chromothripsis and causing 3p loss and 5q gain (Figures 6A and S6). Such events were seen in 15 of 38 (39%) samples, a very similar rate to the 43% we observed in the sporadic cases.

**Figure S7:**
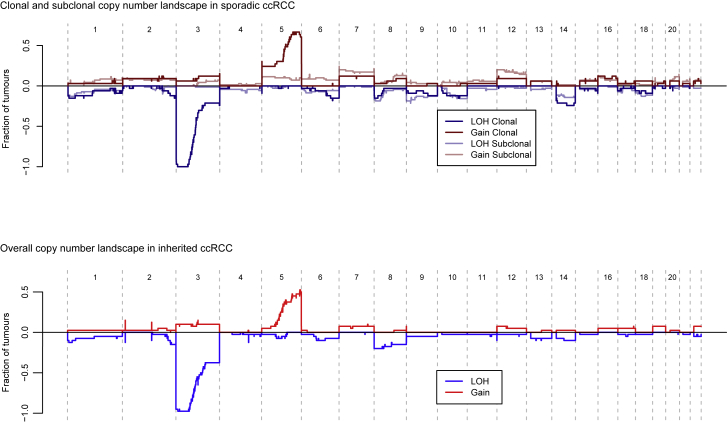
Comparison of the Copy Number Landscape in Sporadic and Inherited (vHL) Datasets, Related to Figure 6

Furthermore, the landscape of copy number aberrations (Figure S7), the trinucleotide context of base-pair substitutions (Figure S3) and distribution of somatic driver mutations in the inherited clear cell renal cell carcinomas was very similar to that seen in sporadic cases. Inactivating mutations were seen in the other key tumor suppressor genes on chromosome 3p, *PBRM1*, *BAP1*, and *SETD2* (Figure 6B). We identified one of the hotspot 5′ UTR mutations in *TERT* in a VHL patient’s tumor (Figure 2A). Furthermore, as reported in the original paper (Fei et al., 2016), the overall burden of mutations increased linearly with age at a similar rate to our estimate in sporadic renal cancers, with similar inter-individual variation (Figure 6C).

We used the same approach described above to estimate the age of chromosome 3p loss in inherited clear cell renal cell carcinomas where the 3p loss was acquired in the same event as a copy number gain (typically 5q) (Figure 6D). As for the sporadic cases, we estimate that the majority of these complex chromosomal rearrangements occur during childhood and adolescence, years to decades before disease diagnosis, with a couple of cases occurring during early adulthood.

Overall, sporadic clear cell renal cell carcinomas and those arising in the context of von Hippel-Lindau disease have remarkably similar evolutionary trajectories and patterns of somatic driver mutations. The major difference is the need to acquire a second somatic *VHL* inactivation event in the sporadic setting.

### Modeling the Early Clonal Dynamics of Sporadic Kidney Cancer

If the major genomic difference between inherited and sporadic clear cell renal cell carcinoma is whether the *VHL* inactivation is germline or somatic, then the difference in age-incidence curves between the two scenarios derives from the time taken to acquire the second *VHL* mutation in the sporadic case. Knowing which mutations in *VHL* are driver mutations and the average rate of these mutations per cell per year, we can estimate how many cells with 3p loss must be present to generate the observed difference in age-incidence curves.

This is a twist on Knudson’s pioneering work leading to the two-hit hypothesis for the then-unknown tumor suppressor gene in retinoblastoma (Knudson, 1971). In his original paper, Knudson (1971) used the known number of retinal ganglion cells to estimate the driver mutation rate in the then-unknown gene from the age-incidence curve of inherited retinoblastoma. He then showed that the age-incidence curve for sporadic retinoblastoma can be reproduced assuming two such mutations are needed at the estimated mutation rate. In our case, we know the target gene, *VHL*, and can directly estimate its rate of driver mutations: what we would like to know is the number of cells at risk after loss of chromosome 3p, namely the size of that initial clonal expansion after deletion of one copy of *VHL*, *PBRM1*, *SETD2*, and *BAP1*.

**Figure 7:**
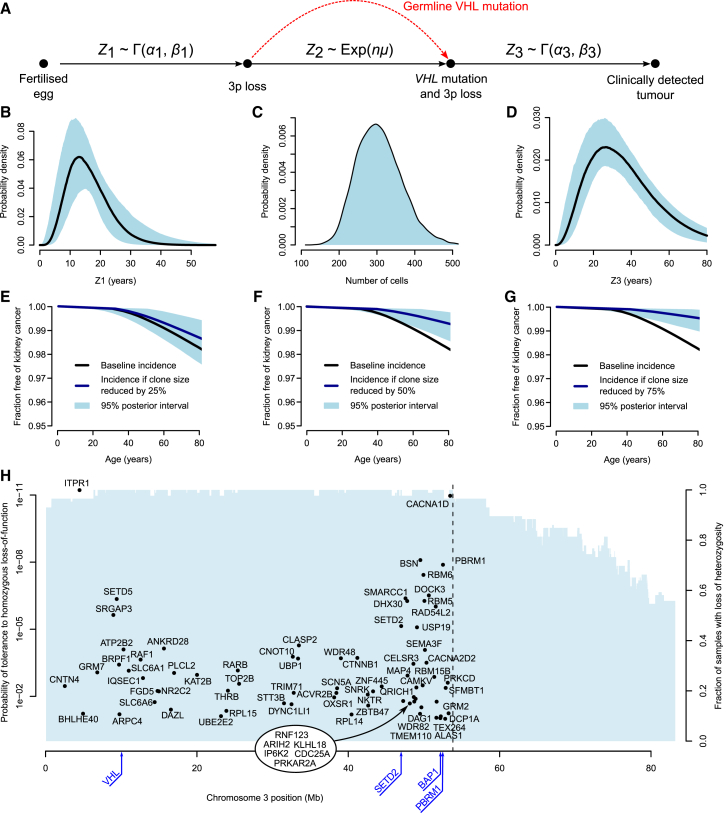
Mathematical Modeling of Clear Cell Renal Cell Carcinoma Evolution (A) Schematic depicting how the age of incidence of renal cell carcinoma may be modeled as the sum of waiting times; *Z*_1_ representing the time to 3p loss, *Z*_2_ representing the time to *VHL* inactivation, and *Z*_3_ representing the time from bi-allelic loss of *VHL* to clinically detected tumor. *Z*_1_ and *Z*_3_ are modeled by gamma distributions and *Z*_2_ by an exponential distribution of the product of *n*, the number of cells with 3p loss and μ, the calculated *VHL* mutational rate. (B–D) The posterior distribution of the waiting times for *Z*_1_ (B), the number of cells with 3p loss (C), and the waiting time for *Z*_3_ (D) with 95% posterior intervals. (E–G) The effect on age-incidence curves for sporadic kidney cancer with reduction of the 3p loss clone size by 25 (E), 50 (F), and 75% (G), with 95% posterior intervals shaded. (H) Location of genes with loss of function intolerance >90% (Lek et al., 2016) that lie within the region of ubiquitous loss in clear cell renal cell carcinoma. The locations of the canonical clear cell tumor suppressor genes are annotated in blue below the x axis.

We used a Bayesian framework to model the published age-incidence curves for inherited (Ong et al., 2007) and sporadic (Cancer Research UK, 2017) clear cell renal cell carcinoma. Briefly, the incidence of inherited clear cell renal cell carcinoma is modeled as the sum of two waiting times: time to 3p loss, estimated from the ages of t(3;5) translocations, plus time from 3p loss to tumor diagnosis (Figure 7A; Data S1; STAR Methods). The incidence of sporadic clear cell renal cell carcinoma is treated as the sum of the same two waiting times plus an additional waiting time for acquisition of a somatic *VHL* driver mutation. This latter waiting time is dependent on the number of susceptible cells, the variable of interest here, and the rate of acquisition of *VHL* driver mutations per year per cell. We directly estimate this from the catalog of mutations in the COSMIC database (Forbes et al., 2015), where we have a reasonably complete description of which point mutations in *VHL* can be drivers of clear cell renal cell carcinoma. These include nonsense, frameshift, splice site and hotspot missense mutations. Given this set of potential drivers, the sequence composition of the gene and the overall mutation rates and signatures observed in our study, we can calculate the rate at which *VHL* driver mutations occur per cell (STAR Methods). This generates an estimate of 2.1 × 10^−6^ driver mutations in *VHL* per year per susceptible cell.

The model generates stable estimates of the key variables (Figures 7B–7D). As intended, the posterior distribution for the waiting time to chromosome 3p loss matches the estimates from the t(3;5) timings (Figure 7B). The waiting time from biallelic *VHL* inactivation to cancer diagnosis ranged from 15 to 30 years (Figure 7D), the wide range presumably reflecting differences in rate of tumor growth, acquisition of subclonal drivers, screening practices, and development of symptoms.

We predict that after chromosome 3p loss in non-carriers, there would only be a few hundred cells with the potential to initiate a future clear cell renal cell carcinoma if a somatic *VHL* mutation were acquired (Figure 7C). It is this population size that best explains the pronounced differences in penetrance and age of incidence between somatic and inherited cancers, given a *VHL* driver mutation rate of ∼2 per million cells per year. This rather modest initial clonal expansion after the first driver event is reminiscent of the limited clonal expansions seen with driver mutations in, for example, normal skin tissue (Martincorena et al., 2015).

### Opportunities for Prevention of Sporadic Clear Cell Renal Cell Carcinoma

The relatively small numbers of cells with chromosome 3p loss that have the future potential to initiate a clear cell renal cell carcinoma, together with the long latency between 3p loss and further progression, suggest a useful therapeutic window in which early intervention could prevent renal cell carcinomas. We used our Bayesian model to simulate the age-incidence curves of sporadic clear cell renal cell carcinoma if the number of cells carrying 3p loss were reduced (Figures 7E–7G). This suggests that we could halve the incidence of sporadic clear cell renal cell carcinoma within the normal human lifespan by reducing the 3p-LOH clone size by 50% (Figure 7F) and have even more profound benefits with more cell kill (Figure 7G).

One of the reasons that this could be such an interesting preventative opportunity is that the region of 3p loss invariably encompasses all four tumor suppressor genes of *VHL*, *PBRM1*, *BAP1*, and *SETD2*, and hence spans at least 40 Mb. There are a large number of genes within this region that have been identified as “essential” to cellular survival in *in vitro* studies (Blomen et al., 2015, Wang et al., 2015) or intolerant of protein-truncating germline mutations *in vivo* (Lek et al., 2016) (Figure 7H). Many of these genes could represent viable therapeutic targets.

## Discussion

Our data reveal that the early development of clear cell renal cell carcinoma follows strongly preferred evolutionary trajectories. Chromosome 3p loss is often the initiating driver, seemingly arising in childhood or adolescence, even though the cancer may not be diagnosed for another 30–50 years. The clonal expansion after 3p loss may not be that large—no more than a few hundred cells with the eventual capability of initiating an invasive cancer. Indeed, these few hundred cells may be distributed across several independent clones and probably exist in all adults—in von Hippel-Lindau disease, where the other *VHL* allele carries a germline mutation, clear cell renal cell carcinoma is nearly completely penetrant and multiple cancers can develop simultaneously (Nielsen et al., 2016). These are clonally unrelated (Fei et al., 2016) and have independent t(3;5) chromothripsis events (Figures 6A and S6). That the first somatic driver mutation would trigger only small clonal expansions has also been suggested by immunohistochemical studies in normal kidney tubules from von Hippel-Lindau disease (Mandriota et al., 2002).

The other critical event, always on the trunk of the phylogenetic tree, is inactivation of the second allele of *VHL*. In all but one patient with informative data here, there was a time lag between the t(3;5) event and the emergence of the most recent common ancestor of the tumor. This suggests that point mutation of *VHL* typically occurs after 3p loss. Sometimes, there is another driver mutation on the trunk of the phylogenetic tree (Gerlinger et al., 2012, Gerlinger et al., 2014), drawn from a range of cancer genes, including *PBRM1*, *SETD2*, *BAP1*, *TERT*, the PI3K signaling pathway and other cytogenetic abnormalities. With a wider repertoire of co-operating genes available, this other truncal driver mutation is considerably less rate-limiting than 3p loss and *VHL* inactivation. Once acquired, these truncal driver mutations trigger a substantial clonal expansion—at this stage, the nascent tumor has a sufficient population size that mutation rate is no longer rate-limiting, which may explain why parallel evolution is so frequently observed in the later stages of renal cancer development (Gerlinger et al., 2012, Gerlinger et al., 2014). Nonetheless, we find there can be a delay of many decades between the emergence of the most recent common ancestor and tumor diagnosis, so although the clonal expansion is substantial, it is not rapid.

There are four key factors recommending 3p loss as a therapeutic target in clear cell renal cell carcinoma: (1) 3p loss is virtually universal in clear cell renal cell carcinoma and is typically the initiating event; (2) the region lost is always large (>40 Mb), because it has to encompass all of *VHL*, *PBRM1*, *SETD2*, and *BAP1*; (3) our data suggest a latency of many decades between 3p deletion and cancer emergence, offering a long therapeutic window in which to deploy an effective therapy; and (4) clonal expansion after 3p loss is not large, and moderate cell kill at this stage would have clinically meaningful impact on cancer incidence.

What could constitute a therapy aimed at 3p loss? Such an agent would not necessarily need to target the four tumor suppressor genes on 3p, nor any of the genes on 5q that are often concurrently gained. Rather, we believe that it is the co-deleted, bystander genes on 3p that might confer the greatest therapeutic vulnerability. Any of the essential genes in Figure 7H might be sufficiently sensitive to gene dosage that a therapeutic agent could have disproportionate effects on cells with 3p deletion. In support of this, several studies published recently have shown that bystander genes can be relevant therapeutic targets in cancers with deletions of specific tumor suppressor genes (Dey et al., 2017, Kryukov et al., 2016, Nijhawan et al., 2012). Such an agent would potentially have efficacy in patients with established clear cell renal cell carcinoma and could have interesting early intervention applications in inherited vHL disease. We provide a thought experiment showing the impact a therapy targeting cells with 3p loss agent could theoretically have as a prevention therapy. At best, though, with a lifetime risk of clear cell renal cell carcinoma of 1%–2% in the sporadic setting, the number-needed-to-treat to prevent one clear cell renal cell carcinoma would be 50–100.

By the time we enter adulthood, all of us will already carry a few hundred seeds with the potential to beget future lethal clear cell renal cell carcinomas. For those of us who have inherited a faulty *VHL* allele, the eventual germination of one or more of these seeds is virtually inevitable within the human lifespan. For the unlucky among the rest of us, that second hit in *VHL* will occur sufficiently quickly that a cancer will develop in middle age or beyond. With an aging and fattening population, the unlucky will nearly double in numbers in 20 years’ time (Smittenaar et al., 2016)—unless, that is, we can harness the long latency, the pre-determined early evolutionary trajectory and the small number of seeds to develop new preventative strategies for renal cancer.

## STAR★Methods

### Key Resources Table

**Table:** 

REAGENT or RESOURCE	SOURCE	IDENTIFIER
**Biological Samples**		

Multi-regional biopsies and blood normal samples from patients with renal cell cancer specimens	Turajlic et al., 2018a	http://tracerx.co.uk/studies/renal/

**Chemicals, Peptides, and Recombinant Proteins**		

PCR buffer	Thermo-Fisher	Cat#CM-251
dNTPs	Thermo-Fisher	Cat#SP-1050
Bitaine	5M	Cat#77507
TAQ polymerase	Thermo-Fisher	Cat#AB-0908
Gel ladder	LONZA	Cat#50473
PCR product cleanup	Thermo-Fisher	Cat#78201.1.ML

**Deposited Data**		

Raw and analyzed data	This paper	EGAD00001003445
Observed mutations in the *VHL* gene	Catalogue Of Somatic Mutations In Cancer	http://cancer.sanger.ac.uk/cosmic/
WGS data from “Patient-specific factors influence somatic variation patterns in von Hippel–Lindau disease renal tumors”	dbGAP	phs001107.v1.p1

**Oligonucleotides**		

Primer CACCCGTCCTGCCCCTTCACCTT	This paper	N/A
Primer CGCAGCCACTACCGCGAGGTGCT	This paper	N/A

**Software and Algorithms**		

CaVEMan	Jones et al., 2016	https://github.com/cancerit/CaVEMan
Pindel	Raine et al., 2015	https://github.com/genome/pindel
BRASS	Campbell et al., 2008	https://github.com/cancerit/BRASS
Battenberg	Nik-Zainal et al., 2012	https://github.com/cancerit/cgpBattenberg
Telomerecat	Farmery et al., 2017	https://pypi.python.org/pypi/telomerecat
N-dimensional clustering of mutations	Nik-Zainal et al., 2012	Available on request
Non-coding driver discovery	Nik-Zainal et al., 2016	https://github.com/im3sanger/dndscv
Estimation of mutation rate per year and ages at which landmark events occur	This paper	Data S1
Rate of VHL driver mutations	This paper	Data S1
Models of age-incidence curves for sporadic & inherited ccRCC	This paper	Data S1

### Contact for Reagent and Resource Sharing

Further information and requests for resources and reagents should be directed to and will be fulfilled by the Lead Contact, Peter J. Campbell (pc8@sanger.ac.uk).

### Experimental Model and Subject Details

Multi-region tumor samples were collected from patients enrolled in TRACERx Renal study (Turajlic and Swanton, 2017) (National Health Service Research Ethics Committee approval 11/LO/1996). The study sponsor is the Royal Marsden NHS Foundation Trust, the chief investigator Dr Samra Turajlic is responsible for study oversight, and the study is coordinated by the Renal Unit at the Royal Marsden Hospital. The TRACERx Renal consortium contributed collectively to this study. Samples were collected at the various study sites and processed by the laboratory at the Francis Crick Institute. Eligible patients were > 18 years with a suspected diagnosis of renal cell cancer of any stage, undergoing resection of the primary tumor. Only cases with clear cell histology at initial histopathological examination were included in the analyses, although one tumor (K169) could not be subsequently classified (Table S2). Detailed study criteria, procedures and sample classification are available in the companion paper (Turajlic et al., 2018a).

### Method Details

#### DNA sequencing and alignment

150 base paired-end sequencing was performed with the HiSeq X Ten system according to Illumina protocols. Average coverage was 67x for tumor samples and 36x for normal samples (Table S1). Alignment of paired-end reads to the reference human genome (GRCh37) used the Burrows-Wheeler Aligner, BWA-MEM.

#### Variant detection

Single-nucleotide substitutions were called using the CaVEMan (cancer variants through expectation maximization) algorithm (https://github.com/cancerit/CaVEMan; Jones et al., 2016). Small insertions and deletions were called using split-read mapping implemented by the Pindel algorithm (https://github.com/genome/pindel; Raine et al., 2015). To call rearrangements we applied the BRASS (breakpoint via assembly) algorithm, which identifies rearrangements by grouping discordant read pairs that point to the same breakpoint event (https://github.com/cancerit/BRASS; Campbell et al., 2008). All mutations were annotated to Ensembl version 58. Copy-number data were derived from whole-genome reads using the Battenberg algorithm (https://github.com/cancerit/cgpBattenberg; Nik-Zainal et al., 2012).

#### Variant validation

We assessed the precision and recall of the whole genome sequencing analysis using the deep, targeted panel sequencing data from multi-region samples reported in the companion paper. Of the 127 somatic driver point mutations reported in Figure 1B, there were 17 discrepancies between the WGS and the panel dataset. These discrepancies break down as follows:

• 9 mutations were not detected in the whole genome sequencing data, but were in the panel data. In all cases, they were present subclonally in the panel data, typically in only a single biopsy. There were no reads reporting these variants in the whole genome sequencing data.
• 5 mutations were called as ‘clonal’ in the whole genome sequencing data but ‘subclonal’ in the panel data. This occurred because mutations had a high variant allele fraction in the WGS data, but were absent from one or more biopsies studied in the panel sequencing data.
• 3 mutations were called in the WGS but not in the panel sequencing. Again, there were no reads reporting these variants in the panel sequencing data, suggesting they are subclonal variants in the tumor as a whole. Manual review of these variants suggested they were genuine somatic mutations, not sequencing artifacts.

None of the regions with coverage < 60x had missed any mutations called in the driver panel. We also found correlation between coverage depth and number of mutations called (r^2^ = 0.20), suggesting that although the obtained sequencing depth was somewhat variable, this had little impact on our variant calling.

Thus, all the discrepancies between the WGS and panel sequencing were due to the spatial heterogeneity of kidney cancers, and the variant calling algorithms appeared to be performing well. A discussion regarding the optimum number of biopsies required for full driver detection takes place in the companion paper; even 10 biopsies will miss the occasional drivers.

#### Capillary sequencing validation of TERT mutations

An additional 286 samples from 94 patients with ccRCC underwent a focal screen of the *TERT* promoter to validate mutations detected in this dataset (Figure 2A). Briefly, the DNA dilutions were prepared at a concentration of approx. 8ng/μl. Primers (CACCCGTCCTGCCCCTTCACCTT and CGCAGCCACTACCGCGAGGTGCT, Sigma-Aldrich) were diluted to a 400 μmol concentration.

The PCR premix was made using for each well using 0.94 μL of 10x PCR buffer (Thermo-Fisher dNTP mix cat no CM-251), 0.94 μL of dNTPs (Thermo-Fisher PCR Buffer 1 cat no SP-1050), 1.13 μL of Bitaine (5M Ultrapure Bitaine, Affymetrix p/n 77507), and 0.09 μL of TAQ polymerase (Thermo-Start TAQ, Thermo-Fisher cat no AB-0908). PCRs were setup using 7.5 μL primer mix, 4.5 μL diluted DNA and 3 μL PCR premix. Plates were sealed, briefly centrifuged and run on the thermocycler (MJ Research Tetrad 2) using the following conditions: 95°C for 8 minutes followed by 40 cycles of 62°C for 2 minutes, 72°C for 2 minutes 30 s, 95°C for 15 s and 72°C for 7 minutes.

4ul of PCR product plus 4ul loading dye were run on 2% agarose gel to confirm PCR success. The remaining PCR product was treated with Exosap to clean up unused nucleotides from the initial PCR. 5ul of this product was added to separate plates containing 5ul either forward or reverse primer dilution (for each primer 405ul of 1 in 10 primer dilution was added to 2295ul DDW). The resulting plates were sealed and submitted for capillary sequencing (Table S4).

#### Inference of chromothripsis

The inference that chromothripsis occurs in a single catastrophic event is extensively discussed in the original paper describing this mutational process (Stephens et al., 2011), and the hallmark features have been developed into formal criteria for its recognition (Korbel and Campbell, 2013). Essentially, the inference depends on demonstrating oscillating copy number states; clustered but randomly oriented rearrangement joins; and the potential to reconstruct a single derivative haplotype that explains all observed copy number changes and rearrangements. These inferences have been critiqued (Kinsella et al., 2014), but the predictions have now been validated by *in vitro* studies that have generated all the diagnostic hallmarks of chromothripsis in a single cell cycle (Zhang et al., 2015).

In our data, the oscillating copy number states can readily be observed in Figures S1 (our data), S2 (TCGA cohort) and S6 (the inherited vHL cohort). The clustered but randomly oriented rearrangement joins is evident from the approximately equal distribution of the four colors of rearrangements in these supplementary figures (purple representing tail-to-head orientation; brown head-to-tail; blue head-to-head and green tail-to-tail). We also provide a complete reconstruction of the derivative chromosome for the simplest event in the cohort in Figure 4A, together with a schematic of how the event occurred (Figure 4B).

Other sequences of events that could theoretically generate the same configuration are implausible. We cannot formally exclude the possibility of a simple t(3;5) unbalanced translocation first, followed by a chromothripsis event, but believe it very unlikely for two reasons. First, it would seem that the main selective advantage to the clone derives from the large-scale copy number changes on 3p and 5q, and not the clustered rearrangements themselves. Thus, if a simple unbalanced t(3;5) occurred first, the necessary copy number changes would already have been achieved, and there would be no additional selective advantage to the chromothripsis event. Second, if the translocation and chromothripsis were decoupled in time, there would be no reason why the location of the chromothripsis cluster would overlap the location of the translocation. However, the chromothripsis is never located in a different portion of the derivative chromosome from the translocation – they always overlap.

The hypothesis that the chromothripsis occurred first on 3p and/or 5q, and was followed by a simple translocation, can be formally excluded. If such a sequence of events occurred, the rearrangements would be all intrachromosomal (isolated to 3p and isolated to 5q) bar the one translocation rearrangement. As can clearly be seen in Figures S1 and S6, there are typically many rearrangements between 3p and 5q within the cluster.

#### Assumption of constant mutation rate

The conclusion that chromosome 3p loss occurs in childhood or adolescence rests on the key inference that the mutation rate in our cohort is constant over time. There are several lines of evidence for this. First, the mutational signatures that are present in our kidney cancers are universal across cancer types (Alexandrov et al., 2013), show a linear correlation with age in most tumor types (Alexandrov et al., 2015) and accumulate steadily with age in normal cells at the same rate as the equivalent cancers (Blokzijl et al., 2016, Welch et al., 2012). This suggests that they are intrinsic mutational processes, acting in all somatic cells steadily throughout life. Second, there is a linear relationship between age and point mutation burden across this cohort, with strikingly similar slopes for the inherited and sporadic cases. Even if we allow for the relationship of age to mutation rate to be concave upward, we still estimate very early timing of the t(3;5) events. Third, signatures indicative of tumor-specific mutational processes are absent from the cohort – there are no signatures, for example, of mismatch repair deficiency, homologous recombination deficiency (Alexandrov et al., 2013) or the aristolochic acid exposure seen in Balkan kidney cancers (Scelo et al., 2014). Such processes often accelerate mutation rates late in tumor evolution (de Bruin et al., 2014, Nik-Zainal et al., 2012), and their absence in these renal cancers is consistent with a more constant mutation rate during disease evolution.

We tested the sensitivity of our age estimates to this assumption using two alternative models. In the first, we included a quadratic term for age, effectively allowing for the mutation rate to parabolically increase with age. This extra parameter did not significantly improve the fit of the relationship between age and mutational burden, and furthermore, the estimated age of 3p loss increased by less than a year. Second, we tested the effects of having an unobserved period of time between the occurrence of the last detectable mutation and tumor diagnosis. The reason for this is that for us to be able to detect a mutation, there has to be a clonal expansion, which will take an undefined amount of time that would not be captured in our initial models. Under a range of values for this unobserved time, the estimated age of t(3;5) events did not increase.

### Quantification and Statistical Analysis

#### Non-coding driver analysis

Detection of non-coding drivers relies upon previously published techniques (Nik-Zainal et al., 2016). The method segments the genome into eight classes of functional regions, which are analyzed separately: exons, core promoters, 5′UTR, 3′UTR, introns, non-coding RNA genes, enhancers, and ultra-conserved regions. The expected number of somatic mutations in a given element is estimated using a model accounting for 192 trinucleotide mutation rates and the sequence composition and length of each element. This estimate is refined using a negative binomial regression with covariates, to improve the estimated rate of the element and infer the extent of neutral variation of the mutation rate across elements. The use of a negative binomial regression treats the number of observed mutations in an element as a Poisson observation with rates being gamma distributed across regions. We used three epigenomic vectors (Lawrence et al., 2013) and the local density of mutations in neighboring non-coding regions as covariates. Known driver genes were excluded from the model to avoid inflating the background model.

A separate analysis was performed for substitutions and small insertions/ deletions. The observed counts for each region were compared to the background rates using a negative binomial test, yielding *P*-values for each region. These *P*-values were combined using Fisher’s method and corrected for multiple testing using FDR (Table S3).

#### Telomere length estimation

To estimate telomere length, we used Telomerecat, a ploidy-agnostic method for estimating telomere length from whole genome sequencing data (https://pypi.python.org/pypi/telomerecat; Farmery et al., 2017). The methodology accurately accounts for both aneuploidy and interstitial telomeric reads, and therefore renders estimations more appropriate in the analysis of cancer genomes. The comparison of telomere lengths between samples relied on linear mixed effect (LME) models to account for subclonal mutations and the non-independence of multiple samples from individual patients.

#### Clustering of mutations

Mutations were clustered using a Bayesian Dirichlet based algorithm as described previously (Bolli et al., 2014, Nik-Zainal et al., 2012). Briefly, the expected number of reads for a given mutation if present in one allelic copy of 100% of tumor cells may be estimated based upon the Battenberg derived tumor cell fraction, the copy number at that locus and the total read-depth. The fraction of cells carrying a given mutation is modeled by a Dirichlet process with an adjustment for the decreased sensitivity in identifying mutations in lower tumor fractions. Mutations were thus assigned to clusters according to calculated fraction of clonality. The hierarchical ordering of these clusters was determined by applying the pigeonhole principle.

#### Gene expression analysis

We investigated the relationship between gene expression and the presence/ mechanism of 5q gain using TCGA RNA sequencing data. Results from the Battenberg (copy number) and Brass (structural variant) analyses of the matched TCGA whole genomes (Figure S2) are used as genomic features for the differential expression analysis. Through this analysis, we aim to determine whether:

1. We can detect increased transcription of genes that are present on the duplicated arm of chromosome 5q;
2. There are any significantly differentially expressed genes in the region of the unbalanced t(3;5) translocation.

Data was downloaded in R via the TCGABiolinks package from Bioconductor. Subsequent analysis was carried out using the R package edgeR.

We initially removed all samples with fewer than a total of 20 million reads, and all genes that do not have greater than 0.5 counts per million values in more than two samples. The common dispersion was then calculated, accounting for the presence or absence of chromosome 5 gains or unbalanced translocations with chromosome 3. Finally, a negative binomial generalized log-linear model using Benjamini-Hochberg correction and FDR < 0.05 was used to detect genes that were significantly differentially expressed.

#### Estimation of mutation rate per year and ages at which landmark events occur

The multiregional aspect to this dataset allows us to analyze mutational burden by phylogenetic branch lengths, providing a more accurate estimate of the mutation rate per year. As an illustration, imagine a tumor with two major subclones that diverged at 50% of molecular time, with the two subclonal lineages accumulating mutations equally and at the same rate as before the most recent common ancestor (MRCA). Then, two thirds of the mutations will be subclonal (one third for each lineage) and one third will arise on the trunk of the phylogenetic tree. Knowing the correct phylogenetic structure allows the MRCA to be accurately placed at 50% molecular time, whereas a naive analysis of clonal versus subclonal mutations may place it at 33% time. For this analysis, we therefore use the phylogenetic trees determined by Bayesian Dirichlet based clustering and the pigeonhole principle in order to explore the relationship between age and mutational clone.

We fit LME models in estimating the mutation rate per year (and to check whether the fit is statistically significant) (Data S1). This is required because the different subclones (branches of the phylogenetic tree) within each patient are not independent (they share at least part of their ancestry). The LME models allow us to manage this within-patient correlation in a statistically appropriate framework. We can also generate estimates of the mutation rate per year for each patient specifically, estimates that represent a compromise between the observed rate for each patient and the population average. These models also show that including intercepts do not improve the statistical fit. To explore the possibility of the mutational rate increasing with age, we included a quadratic term for age in the LME model and ran this modified model through the algorithms described below.

To time the onset of landmark events, we fit LME models to estimate age from the number of mutations. We can therefore estimate events such as emergence of the most recent common ancestor (MRCA) and copy number gains (especially the t(3;5) events).

The approach used is to fit the LME, and then use the patient-specific estimates of the slope to time the events from the observed number of mutations that have accumulated by the time that event occurs in that patient. For timing the MRCA, this is simply the number of mutations that are fully clonal, as estimated by the Hierarchical Dirichlet process. For timing the t(3;5) event and other copy number gains that are fully clonal, this can be approached in two ways. First, it can be expressed as a fraction of time between 0 and emergence of MRCA through the fraction of mutations that are duplicated versus clonal but present on only a single copy of the duplicated chromosome (Nik-Zainal et al., 2012). Second, we can estimate directly from the number of mutations present pre-duplication, assuming we know what fraction of the genome has sufficient coverage for calling events. Parametric boot-strapping is used to generate 95% confidence intervals for the timing estimates.

To explore the data, we extract the somatically acquired base substitutions in the duplicated region of chromosome 5q. The variant allele fraction for each mutation called in any sample is extracted and then converted to a cancer cell fraction (CCF; the fraction of cancer cells in the sample that carry the mutation) using the level of normal cell contamination and copy number at that position. From this, we calculate whether the mutation was acquired prior to the duplication of chromosome 5q (if it was, the CCF will be close to 2; if not, CCF ∼ = 1). For clonal mutations (seen in all samples), we take the consensus across all samples to vote whether it more likely occurred before or after chromosome 5q duplication. Results are then plotted for each sample to allow assessment of consistency of the calls/ data (Figures 5B and S4).

We apply two similar methods to estimate the age of occurrence of the t(3;5) gain:

(1) The first is to use the number of chromosome 5q mutations that are clonal and acquired before the 5q duplication relative to the number that are clonal but acquired after duplication (correcting for the fact that post-duplication there is an extra copy of 5q and hence the mutation burden accumulates more quickly). The fraction of clonal time at which the duplication occurred is then estimated from the estimate of when the MRCA emerged. Bootstrapping provides 95% confidence intervals, incorporating the uncertainty in the numbers of pre- and post-duplication mutations and the age the MRCA emerges (Figures 5C, 6D, and S5).
(2) The second method is to derive the age of occurrence directly from the mutation rate estimated per patient from the LME and the number of mutations that have accumulated before the 5q was duplicated. This requires correction for the size of the region gained and what the total size of the genome that could have had mutations called (from the BAM files, we estimate that this is 5.32Gb for a typical sample in this series). Again, bootstrapping provides 95% confidence intervals, incorporating the uncertainty in the numbers of pre-duplication mutations and the patient-specific mutation rate.

#### Rate of VHL driver mutations

To estimate the average rate of driver mutations in *VHL*, we estimate separately the rate of substitutions and indels. To estimate the rate of driver substitutions, we calculate the estimated rate of each of the 6 mutation types in each of the trinucleotide contexts from the overall substitution rate and the observed sequence context of kidney cancer point mutations (Data S1). We then take the length and sequence composition of the coding DNA sequence (CDS) of *VHL* and generate all possible substitutions, and from this extract the set of all possible amino acid consequences arising from substitutions along the length of *VHL*. From this, we define the set of all possible driver substitutions as any substitutions that are: start-lost, stop-lost, stop-gained or a member of the set of previously observed amino acid substitutions in *VHL* recorded in clear cell renal cell carcinomas in the COSMIC database. The rates of these individual mutations are then summed to generate the overall driver substitution rate of 8.5e-07 /cell/year per cell per year.

Estimating the rate of driver indels in *VHL* follows broadly the same approach. We show that there is a strongly linear association between the number of substitutions and the number of indels across patients in the cohort. Using the slope of this relationship, we estimate the indel rate per year per clone from the average substitution rate. We then assume that all indels within the CDS of *VHL* are driver mutations. This then allows us to calculate the *VHL* driver indel mutation rate of 1.2e-06 /cell/year.

#### Models of age-incidence curves for sporadic & inherited ccRCC

We have shown that in this study and companion papers (Turajlic et al., 2018a, Turajlic et al., 2018b), the evolution of sporadic clear cell kidney cancer appears to follow well-defined and recurrent trajectories. Frequently, it seems the first event is chromosome 3p loss, often with concomitant gains on other chromosomes – this appears to occur predominantly in childhood or adolescence. The other key event that occurs early in the evolution of sporadic ccRCC is inactivation of the other allele of *VHL*, typically through point mutation (notwithstanding the role of epigenetic silencing) – this is an obligatory early event, because it is both highly recurrent across patients (> 75%) and always present on the trunk of the phylogenetic tree.

Furthermore, exploring the genomic features of ccRCC that have occurred in the setting of inherited *VHL* mutations reveals many similarities with sporadic ccRCC, (Fei et al., 2016) and analyzed further here. Large-scale chromosomal loss of the other allele of chromosome 3p is universal. The other driver mutations occur in the same genes and at broadly the same frequencies. The mutation rate is similar to that seen in sporadic cancers, with an almost identical spectrum and linear association with age.

We therefore built Bayesian models of the age-incidence curves for sporadic and inherited ccRCC. The first step is to interpret the published age-incidence figures (Data S1). The age-incidence curves for sporadic clear cell RCC come from Cancer Research UK and represent age-specific annual incidence figures per 100,000 population, banded in 5-year groups. The figures for inherited vHL disease are Kaplan-Meier curves derived from (Ong et al., 2007). The second step is to take draws of cohorts of individual patients (or censored non-patients) from the published curves. These then represent the data that is fitted by the Bayesian model.

The primary question of interest is how large is the clone(s) carrying 3p loss during the development of sporadic kidney cancer. From the difference in age-incidence curves between *VHL* carriers and sporadic kidney cancers, and the known *VHL* driver mutation rate per cell per year, we can estimate how many cells that are susceptible to initiating ccRCC carry chromosome 3p loss during adulthood.

The major assumption in this approach is that the evolution of sporadic kidney cancers and inherited ccRCC in vHL patients is identical except for the need to inactivate *VHL* as a somatic event in the sporadic cases. The broad concept for modeling the age-incidence curves is to treat sporadic ccRCC as the sum of three independent waiting times (time to 3p loss; time from 3p loss to *VHL* inactivation; time from biallelic *VHL* loss to diagnosed kidney cancer, Figure 7A). We treat ccRCC in carriers of *VHL* as the sum of two independent waiting times, with the same distribution as in sporadic cases (time to 3p loss; time from 3p loss to diagnosed kidney cancer).

Formally, we let $Y_{i,spor}$ denote the age of incidence (in years) of patient i with sporadic ccRCC, and $Y_{i,vHL}$ the age of incidence of ccRCC in patients with von Hippel-Lindau disease. We then let:

$$Y_{i,spor}=Z_{1}+Z_{2}+Z_{3}$$

$$Y_{i,vHL}=Z_{1}+Z_{3,}$$

where $Z_{1}$ is the waiting time to 3p loss; $Z_{2}$ is the waiting time between 3p loss and somatic inactivation of the other *VHL* allele; and $Z_{3}$ is the waiting time from biallelic *VHL* loss to diagnosis of clear cell renal cancer. Clearly, we do not observe all waiting times, since ∼20% of vHL cases do not develop ccRCC and the vast majority of non-carriers do not. Thus, there will be censoring of the sum of waiting times for many individuals, which we handle by data augmentation.

We model these waiting times with the gamma distribution for $Z_{1}$ and $Z_{3}$and the exponential distribution for $Z_{2}$. That is:

$$Z_{1}∼\text{Γ}\left(\alpha_{1},\beta_{1}\right)$$

$$Z_{2}∼Exp\left(\lambda \right)$$

$$Z_{3}∼\text{Γ}\left(\alpha_{3},\beta_{3}\right),$$

where λ=νμ, with ν as as the number of cells in the clone after chromosome 3p loss and μ as the *VHL* driver mutation rate per cell per year. We use the conjugate prior: λ∼Γ(0.01,0.01). The parameters, $\alpha_{1},\beta_{1},\alpha_{3}\;\mathrm{and}\;\beta_{3}\text{,}$ we model as coming from the conjugate prior to the gamma distribution:

$$\left(\alpha ,\beta \right)\propto \frac{p^{\alpha -1}e^{-\beta q}}{\text{Γ}{\left(\alpha \right)}^{r}\beta^{-\alpha s}}\text{,}$$

where p,q,rands are hyperparameters. For $\alpha_{3}\;and\;\beta_{3}\text{,}$ we use uninformative hyperparameters $\left(p_{3}=q_{3}=r_{3}=s_{3}=1\right)\text{,}$ but for $\alpha_{1}\;\mathrm{and}\;\beta_{1}\text{,}$ we instead choose an informative prior distilled from the estimated ages of chromosome 3p loss. That is,

$$p_{1}=\prod_{i}x_{3p,i};\;q_{1}=\sum_{i}x_{3p,i}\;;\;r_{1}=s_{1}=n_{3p},$$

where $x_{3p,i},\;i=1,\ldots ,n_{3p},$ are the estimated ages at which chromosome 3p loss occurred in patients with informative t(3;-) translocations.

We take draws from the posterior distribution using a Gibbs sampler. We use 50,000 iterations with the first 10,000 being treated as burn-in. The steps involved are as follows:

##### 1. Update $z_{i,1}$ and $z_{i,3}$ for vHL patients who were not censored

Since patients who were not censored have an exact observed age of incidence, $y_{i}\text{,}$ we must take draws such that $z_{i,1}+z_{i,3}=y_{i}\text{.}$ We do this by using a Metropolis-Hastings approach where the proposal distribution is a Dirichlet distribution, scaled to the age of incidence. That is, the proposed new values are:

$$\left(z_{i,1}^{\left(*\right)},z_{i,3}^{\left(*\right)}\right)∼y_{i}\;.\;Dir\left(\frac{\kappa z_{i,1}^{\left(j-1\right)}}{z_{i,1}^{\left(j-1\right)}+z_{i,3}^{\left(j-1\right)}},\frac{\kappa z_{i,3}^{\left(j-1\right)}}{z_{i,1}^{\left(j-1\right)}+z_{i,3}^{\left(j-1\right)}}\right),$$

where κ is a user-defined scaling factor chosen to optimize the acceptance ratio of the Metropolis-Hastings algorithm and the (j−1) superscript denotes the current value of the two waiting times.

The importance ratio for the Metropolis-Hastings algorithm is therefore:

$$\begin{array}{c} Q_{i}=\frac{f\left(z_{i}^{\left(j-1\right)}|z_{i}^{\left(*\right)}\right)}{f\left(z_{i}^{\left(*\right)}|z_{i}^{\left(j-1\right)}\right)}\;.\;\frac{P\left(z_{i}^{\left(*\right)}|z_{i}^{\left(j-1\right)},\alpha_{1}^{\left(j-1\right)},\;\beta_{1}^{\left(j-1\right)},\alpha_{3}^{\left(j-1\right)},\beta_{3}^{\left(j-1\right)}\right)}{P\left(z_{i}^{\left(j-1\right)}|z_{i}^{\left(*\right)},\alpha_{1}^{\left(j-1\right)},\;\beta_{1}^{\left(j-1\right)},\alpha_{3}^{\left(j-1\right)},\beta_{3}^{\left(j-1\right)}\right)}\\ =\frac{f\left(z_{i}^{\left(j-1\right)}|z_{i}^{\left(*\right)}\right)}{f\left(z_{i}^{\left(*\right)}|z_{i}^{\left(j-1\right)}\right)}\;.{\left(\;\frac{z_{i,1}^{\left(*\right)}}{z_{i,1}^{\left(j-1\right)}}\right)}^{\alpha_{1}^{\left(j-1\right)}-1}.\;{\left(\;\frac{z_{i,3}^{\left(*\right)}}{z_{i,3}^{\left(j-1\right)}}\right)}^{\alpha_{3}^{\left(j-1\right)}-1}.e^{-\beta_{1}^{\left(j-1\right)}\left(z_{i,1}^{\left(*\right)}-z_{i,1}^{\left(j-1\right)}\right)-\beta_{3}^{\left(j-1\right)}\left(z_{i,3}^{\left(*\right)}-z_{i,3}^{\left(j-1\right)}\right)}. \end{array}$$

This is accepted or rejected in the usual way.

##### 2. Update of $z_{i,1}$and $z_{i,3}$ for vHL patients who were censored

Here, we know only the lower bound on the $y_{i}$, so we sample these using rejection sampling. That is, we take draws of $z_{i,1}^{\left(*\right)}∼\text{Γ}\left(\alpha_{1}^{\left(j-1\right)},\beta_{1}^{\left(j-1\right)}\right)$ and $z_{i,3}^{\left(*\right)}∼\text{Γ}\left(\alpha_{3}^{\left(j-1\right)},\beta_{3}^{\left(j-1\right)}\right)$ until $z_{i,1}^{\left(*\right)}+z_{i,3}^{\left(*\right)}\;$is greater than the age of censoring.

##### 3. Update of $z_{i,1},z_{i,2}$and $z_{i,3}$ for sporadic ccRCC patients who were not censored

We apply the same approach as in step 1, using an analogous three parameter Dirichlet proposal distribution.

##### 4. Update of $z_{i,1},z_{i,2}$and $z_{i,3}$ for sporadic ccRCC patients who were censored

We apply the analogous rejection sampling approach as used in step 2.

##### 5. Update of $\alpha_{1},\beta_{1},\alpha_{3},\beta_{3}$ from conjugate prior

Given the (informative) hyperparameters $p_{1},q_{1},r_{1},\;s_{1}$for the conjugate prior for $\left(\alpha_{1},\beta_{1}\right)\text{,}$ we have:

$$f\left(\alpha_{1},\beta_{1}|p_{1},q_{1},r_{1},s_{1}\right)\propto \;\frac{{\left(p_{1}\prod_{i}z_{i,1}^{\left(j\right)}\right)}^{\alpha_{1}}\;.\;e^{-\beta_{1}\left(q_{1}+\sum_{i}z_{i,1}^{\left(j\right)}\right)}}{\text{Γ}{\left(\alpha_{1}\right)}^{r_{1}+n}\;.\;\beta_{1}^{-\alpha_{1}\left(s_{1}+n\right)}}.$$

We sample from this using a Metropolis-Hastings algorithm with the proposal distribution as independent gamma variables: $\alpha_{1}^{\left(*\right)}∼\text{Γ}\left(\alpha_{1}^{\left(j-1\right)}\gamma \right)$ and $\beta_{1}^{\left(*\right)}∼\text{Γ}\left(\beta_{1}^{\left(j-1\right)}\gamma \right)$, where γ is a scaling factor defined by the user to optimize the acceptance / rejection ratio. The importance ratio is calculated, and the proposed values, $\alpha_{1}^{\left(*\right)}$ and $\beta_{1}^{\left(*\right)}\text{,}$ accepted or rejected in the usual way. The same approach is applied to updating $\alpha_{3}$ and $\beta_{3}$.

##### 6. Update of λ

We take draws directly from the posterior:

$$\lambda^{\left(j\right)}∼\text{Γ}\left(0.01+n_{spor}\;,\;0.01+\sum_{i}z_{i,2}^{\left(j\right)}\right)\text{.}$$

##### 7. Draws from posterior distribution of ages of incidence under different clone sizes

We can also take draws of alternative $z_{i,2}$ values if the clone size were different, by a fraction ρ. That is, we draw $z_{i,2}^{\left(j\right)}∼Exp\left(\rho \lambda^{\left(j\right)}\right)$ and add to $z_{i,1}^{\left(j\right)}+z_{i,3}^{\left(j\right)}$ to generate an alternative age of incidence.

There are a few points of note in this implementation of the model. First, we assume independence of the waiting times. If, for example, there is clone-to-clone variation in the mutation rate, this assumption may not be entirely valid, since the time from chromosome 3p loss to *VHL* inactivation would potentially be correlated with the time from *VHL* inactivation to kidney cancer resection. However, it is unclear how much clone-to-clone variation there is in mutation rate among kidney cells and how much inter-individual variation. Second, we assume that the clone size is broadly constant after chromosome 3p loss. That is, the clone expands rapidly to a steady-state number, at which stage it plateaus. In fact, given the relatively small number of cells estimated in the clones, this is probably a reasonable assumption. Third, we do make the assumption that the order of events is chromosome 3p loss, followed by *VHL* point mutation, followed by other driver mutations, clonal expansion and diagnosis. Given that we are estimating a clone size of several hundred cells after chromosome 3p loss, it is statistically much more likely that the *VHL* mutation will occur after this clonal expansion than in the one cell before chromosome 3p loss (inherent in this is the assumption that the rate of chromosome 3p loss through, for example, chromothripsis involving chromosomes 3p and 5q is much lower than that of *VHL* driver point mutations).

We check some convergence and mixing plots for the α and β estimates. They show rather slow mixing for $\alpha_{1}$ and $\beta_{1}$. This is perhaps not surprising since the information for the $Z_{1}$ waiting time is somewhat confounded with the $Z_{3}$ waiting time, except through the prior information provided from the timings of t(3;-) translocations. Nonetheless, we have experimented with many different starting points for these values, and the posterior always converges well to the distribution (Figures 7B and 7D).

The key question of interest in this model is to establish the potential number of cells that carry chromosome 3p loss without having the other *VHL* allele mutated. The MCMC draws from the posterior distribution converge and mix well. From the posterior distribution it is suggested that the number of cells in the kidney that carry chromosome 3p loss before the other VHL allele is mutated is only in the hundreds (Figure 7C).

The other question of interest is to model what would happen to the age-incidence curves for sporadic kidney cancer if we had a treatment that could kill a fraction of cells at the chromosome 3p loss stage. To answer this question, we use the posterior distribution for the sum of waiting times using a depleted number of cells with chromosome 3p loss (Figures 7E–7G).

### Data and Software Availability

The accession number for the genome sequence data reported in this paper is European Genome-Phenome Archive: EGAD00001003445. Mathematical detail, code, and worked examples for the estimation of mutation rate per year and ages at which landmark events occur, the rate of *VHL* driver mutations, and the models of age-incidence curves for sporadic & inherited ccRCC are available in Data S1.

## Consortia

The members of the TRACERx Renal consortium are Tim O’Brien, David Nicol, Ben Challacombe, Archana Fernando, Steve Hazell, Ashish Chandra, James Larkin, Martin Gore, Lisa Pickering, Sarah Rudman, Simon Chowdhury, Karen Harrison-Phipps, Mary Varia, Catherine Horsfield, Alexander Polson, Gordon Stamp, Marie O’Donnell, William Drake, Peter Hill, David Hrouda, Eric Mayer, Jonathon Olsburgh, Gordon Kooiman, Kevin O’Connor, Grant Stewart, Michael Aithcison, Maxine Tran, Nicos Fotiadis, Hema Verma, and Jose I. Lopez.Acknowledgments

## Supplemental Information

Table S1. Sequencing and Sample Metrics for Individual Biopsies Included in Study, Related to Figure 1

Table S2. Clinical Characteristics and Burden of Unique Somatic Variants by Patient for All Tumors that Underwent WGS, Related to Figure 1

Table S3. Significantly Recurrent Mutations in Coding and Non-coding Regions, Related to Figure 2

Table S4. Detection of Additional TERT Promoter Mutations in Validation Cohort, Related to Figure 2

Table S5. Telomere Length Estimation from WGS Data, Related to Figure 2

Table S6. Differential Expression of Genes on Chromosome 5 Related to Chromosomal Gain, Related to Figure 3

Data S1. R Code for Timing Analysis, Related to Figure 5 Interwoven R code, discussion and results for analysis of molecular and chronological timing of landmark events in ccRCC evolution. The document was created using the ‘knitr’ package.
