# Supplementary material for: Timing the Landmark Events in the Evolution of Clear Cell Renal Cell Cancer: TRACERx Renal
Source: Cell. 2018 Apr 19;173(3):611–623.e17. doi: 10.1016/j.cell.2018.02.020 (PMC5927631; doi:10.1016/j.cell.2018.02.020)
Supplement: Data S1. R Code for Timing Analysis, Related to Figure 5 — Interwoven R code, discussion and results for analysis of molecular and chronological timing of landmark events in ccRCC evolution. The document was created using the ‘knitr’ package. [file mmc7.zip › Supplemental Code/Mathematical modeling of the evolution of kidney cancer.html]

Supplementary Material


# Supplementary Material

# Analysis of timing of MRCA & copy number gains

knitr document to capture the methods and R code for analysing chronological age at which landmark events occur during kidney cancer development.

## Mutation burden as a molecular clock

The analyses here are predicated on the assumption that total mutation burden in kidney cancer can act as a molecular clock. There are three major lines of evidence supporting this, discussed further in the main manuscript:

(1) Overall mutation burden in kidney cancer correlates linearly with age at diagnosis (explored further below);

(2) Mutation signatures in kidney cancer are mostly restricted to signatures 1 and 5, signatures that are nearly
ubiquitous across tumour types and show a linear correlation with age
(see Alexandrov et al, Nature Genetics 2015 );

(3) In normal cells of blood and other tissues, the burden of mutations attributed to signatures 1 and 5
is broadly equivalent to tumours from people of the same age
(see, for example, Welch et al, Cell 2012).

To explore the relationship between total mutation burden and age in this dataset, we have the advantage
that we can analyse by phylogenetic branch lengths, providing a more accurate estimate of the mutation rate per year.
To see this, imagine a tumour with two major subclones that diverged at 50% of molecular time, with the two subclonal
lineages accumulating mutations equally and at the same rate as before the most recent common ancestor (MRCA).
Then, two thirds of the mutations will be subclonal (one third for each lineage) and one third will arise on the trunk
of the phylogenetic tree. Knowing the correct phylogenetic structure allows the MRCA to be accurately placed at
50% molecular time, whereas a naive analysis of clonal versus subclonal mutations may place it at 33% time.

The first analysis is to read in the data files and explore the relationship between age and mutation burden per branch
of the phylogenetic tree.

```
knitr::opts_chunk$set(fig.width=12, fig.height=8, warning=FALSE)

library(ggplot2)
library(reshape2)
library(MASS)
library(lme4)
```

```
## Loading required package: Matrix
```

```
library(pander)
library(survival)
library(MCMCpack)
```

```
## Loading required package: coda
```

```
## ##
## ## Markov Chain Monte Carlo Package (MCMCpack)
```

```
## ## Copyright (C) 2003-2017 Andrew D. Martin, Kevin M. Quinn, and Jong Hee Park
```

```
## ##
## ## Support provided by the U.S. National Science Foundation
```

```
## ## (Grants SES-0350646 and SES-0350613)
## ##
```

```
library(extraDistr)
```

```
## 
## Attaching package: 'extraDistr'
```

```
## The following objects are masked from 'package:MCMCpack':
## 
##     ddirichlet, dinvgamma, rdirichlet, rinvgamma
```

```
library(abind)

# Set-up some basic script parameters
if (!exists("rcc.iters")) { rcc.iters <- 50000 }        # number of iterations for the Monte Carlo Markov chain (MCMC)
if (!exists("rcc.burn.in")) { rcc.burn.in <- 20000 }     # number of burn ins for the MCMC
if (!exists("chrono.seed"))  { chrono.seed <- 31 }

set.seed(chrono.seed)

print(rcc.iters)
```

[1] 50000

```
print(rcc.burn.in)
```

[1] 20000

```
print(chrono.seed)
```

[1] 31

```
# set.seed(223)
# set.seed(28)
# set.seed(14)
# set.seed(88)
# rcc.iters <- 50000
# rcc.burn.in <- 20000    

# Read in patient data
pt.df <- read.table("../../data/summary/patient_summary.txt", sep="\t", header=TRUE, stringsAsFactors = FALSE)
pt.df <- pt.df[pt.df$include == "yes",]

# Read in phylogenetic trees
branch.df <- data.frame(Sample=character(0), Name=character(0), Num.clonal=integer(0), Num.mutations=integer(0))

branch.files <- system("ls params/Tree_structures/*optima*", intern = TRUE)
branch.files <- branch.files[substring(branch.files, first = 24, last = 30) %in% pt.df$sanger.ID]
branch.files <- branch.files[!(substring(branch.files, first = 24, last = 30) %in% c("PD24238", "PD24242"))] 
# This previous line removes a patient for which only one sample gave good sequence; and a tumour with two completely independent clones (ie two separate cancers)

for (i in branch.files)
{
  temp <- suppressWarnings(read.table(i, header=TRUE, sep="\t", stringsAsFactors = FALSE))
  sample <- substring(i, 24, 30)
  lri.sample <- pt.df[pt.df$"sanger.ID"==sample,2]

  temp.term <- data.frame(Sample = rep(sample, sum(temp$Terminal == "Yes")), 
                          LRISample = rep(lri.sample, sum(temp$Terminal == "Yes")), 
                          Name = temp$Name[temp$Terminal == "Yes"],
                          Num.clonal = rep(temp$no.of.mutations.assigned[temp$Name=="T"], sum(temp$Terminal=="Yes")),
                          Num.mutations = rep(0, sum(temp$Terminal == "Yes")),
                          Age = rep(pt.df$Age[pt.df$sanger.ID == sample], sum(temp$Terminal == "Yes")))

  # This next loop calculates the number of mutations from root of phylogenetic tree to tip of each terminal branch
  for (j in temp.term$Name)
  {
    constituents <- unlist(strsplit(j, ">"))
    for (k in 2:length(constituents)) {constituents[k] <- paste(constituents[k-1], constituents[k], sep=">")}
    temp.term$Num.mutations[temp.term$Name == j] <- sum(temp$no.of.mutations.assigned[temp$Name %in% constituents])
  }
  branch.df <- rbind(branch.df, temp.term)  
}

# Now plot the relationship between number of mutations on each branch and age
ggplot(data = branch.df, aes(x=Age, y=Num.mutations, color=LRISample)) + 
  geom_point(shape=16) +
  xlim(c(0,80)) + ylim(c(0,10000)) +
  xlab("Age (years)") + ylab("Number of mutations per subclone")
```

## Fit linear mixed effects models to estimate mutation rate per year

To enable the estimation of the mutation rate per year (and check whether the fit is statistically significant),
we fit linear mixed effects (LME) models. This is required because of course the different subclones
(branches of the phylogenetic tree) within each patient are not independent (they share at least part of their ancestry).
The LME models allow us to manage this within-patient correlation in a statistically appropriate framework.
We will also be able to generate estimates of the mutation rate per year for each patient specifically,
estimates that represent a compromise between the observed rate for each patient and the population average.

```
# Test fits with and without intercepts

muts.per.year.lmer.with.pt.intercepts <- lmer(Num.mutations ~ Age + (Age | Sample), data=branch.df, REML=FALSE)
muts.per.year.lmer.with.pop.intercept <- lmer(Num.mutations ~ Age -1 + (Age | Sample), data=branch.df, REML=FALSE)
muts.per.year.lmer <- lmer(Num.mutations ~ Age - 1 + (Age - 1 | Sample), data=branch.df, REML=FALSE)

kable(anova(muts.per.year.lmer, muts.per.year.lmer.with.pop.intercept, muts.per.year.lmer.with.pt.intercepts))
```

|  | Df | AIC | BIC | logLik | deviance | Chisq | Chi Df | Pr(>Chisq) |
| --- | --- | --- | --- | --- | --- | --- | --- | --- |
| muts.per.year.lmer | 3 | 1241.274 | 1248.187 | -617.6372 | 1235.274 | NA | NA | NA |
| muts.per.year.lmer.with.pop.intercept | 5 | 1245.138 | 1256.659 | -617.5692 | 1235.138 | 0.1360513 | 2 | 0.9342365 |
| muts.per.year.lmer.with.pt.intercepts | 6 | 1247.080 | 1260.905 | -617.5401 | 1235.080 | 0.0580850 | 1 | 0.8095487 |

```
# Print summary of model without intercepts
print(summary(muts.per.year.lmer))
```

Linear mixed model fit by maximum likelihood ['lmerMod']
Formula: Num.mutations ~ Age - 1 + (Age - 1 | Sample)
Data: branch.df

```
 AIC      BIC   logLik deviance df.resid
```

1241.3 1248.2 -617.6 1235.3 71

Scaled residuals:
Min 1Q Median 3Q Max
-2.2853 -0.4185 0.0333 0.3143 3.1838

Random effects:
Groups Name Variance Std.Dev.
Sample Age 266.9 16.34  
Residual 504921.4 710.58  
Number of obs: 74, groups: Sample, 31

Fixed effects:
Estimate Std. Error t value
Age 87.213 3.261 26.75

```
 # Generate the bootstrap dataset for this LME
 mySumm.muts.per.yr <- function(.) {
   unlist(fixef(.) + ranef(.)$Sample[,1]) 
 }

 boot2 <- bootMer(muts.per.year.lmer, mySumm.muts.per.yr, nsim=1000, use.u=TRUE, type="parametric")
 colnames(boot2$t) <- row.names(ranef(muts.per.year.lmer)$Sample)
```

Thus, there is no evidence that including intercepts helps the fit of the model.

## Fit linear mixed effects models to estimate age at which landmark events occur

### Most recent common ancestor (MRCA)

We now fit linear mixed effects models to estimate age from the number of mutations.
This will then be used to provide estimates of the age at which key landmark events occur,
such as emergence of the most recent common ancestor (MRCA) and copy number gains (especially the t(3;5) events).

The method is to fit the LME, and then use the patient-specific estimates of the slope to time the events
from the observed number of mutations that have accumulated by the time that event occurs in that patient.
For timing the MRCA, this is simply the number of mutations that are fully clonal, as estimated by the Hierarchical
Dirichlet process. For timing the t(3;5) event and other copy number gains that are fully clonal, this can be approached
in two ways. Firstly, it can be expressed as a fraction of time between 0 and emergence of MRCA through the fraction
of mutations that are duplicated versus clonal but present on only a single copy of the duplicated chromosome
(see Nik-Zainal et al, Cell 2012; 149(5): 994-1007
for more details). Secondly, we can estimate directly from the number of mutations present pre-duplication,
assuming we know what fraction of the genome has sufficient coverage for calling events.

Parametric boot-strapping is used to generate 95% confidence intervals for the timing estimates.

```
branch.df$Scaled.num.muts <- scale(branch.df$Num.mutations, center=FALSE)
scale.factor <- attr(branch.df$Scaled.num.muts, which = "scaled:scale")

age.lmer <- lmer(Age ~ Scaled.num.muts - 1 + (Scaled.num.muts - 1 | Sample), data=branch.df)
branch.df$Pt.fitted.num.muts <- scale.factor / (ranef(age.lmer)$Sample[branch.df$Sample,] + fixef(age.lmer)["Scaled.num.muts"]) * branch.df$Age

# Visual check of model's fit
ggplot(data = branch.df, aes(x=Age, y=Num.mutations, color=LRISample)) + 
  geom_segment(aes(x=0, y = 0, xend=Age, yend=Pt.fitted.num.muts, colour=LRISample), data=branch.df) +
  geom_abline(intercept=0,slope=scale.factor / fixef(age.lmer), size=3) +
  geom_point(shape=16) +
  xlim(c(0,80)) + ylim(c(0,10000))
```

```
# Generate point estimates for the timing of the MRCA emergence
new.dat <- data.frame(Sample=branch.df$Sample, LRISample=branch.df$LRISample, Scaled.num.muts=branch.df$Num.clonal / scale.factor, Age=branch.df$Age)
new.dat <- new.dat[!duplicated(new.dat$Sample),]
new.dat$MRCA.pred.age <- predict(age.lmer, newdata = new.dat)

# Now generate 95% CIs on the predictions for MRCA timing
# Note that CIs for lme models are challenging - bootstrapping appears to be most robust

mySumm <- function(.) {
  predict(., newdata=new.dat, re.form=NULL)
}

####Collapse bootstrap into median, 95% PI
sumBoot <- function(merBoot) {
  return(
    data.frame(fit = apply(merBoot$t, 2, function(x) as.numeric(quantile(x, probs=.5, na.rm=TRUE))),
               lwr = apply(merBoot$t, 2, function(x) as.numeric(quantile(x, probs=.025, na.rm=TRUE))),
               upr = apply(merBoot$t, 2, function(x) as.numeric(quantile(x, probs=.975, na.rm=TRUE)))
    )
  )
}

boot1 <- bootMer(age.lmer, mySumm, nsim=1000, use.u=FALSE, type="parametric")

PI.boot1 <- sumBoot(boot1)
PI.boot1$Age <- new.dat$Age
PI.boot1$pred.fit <- new.dat$MRCA.pred.age
PI.boot1$Time.lag <- PI.boot1$Age - PI.boot1$fit
PI.boot1$Time.lag[PI.boot1$Time.lag < 0] <- PI.boot1$Age[PI.boot1$Time.lag < 0] - PI.boot1$pred.fit[PI.boot1$Time.lag < 0]
PI.boot1$lag.lwr <- PI.boot1$Age - PI.boot1$upr
PI.boot1$lag.lwr[PI.boot1$lag.lwr < 0] <- 0
PI.boot1$lag.upr <- PI.boot1$Age - PI.boot1$lwr
PI.boot1$ID <- new.dat$Sample
PI.boot1$LRIID <- new.dat$LRISample
PI.boot1 <- PI.boot1[order(PI.boot1$Time.lag),]

plot(PI.boot1$Time.lag, 1:nrow(PI.boot1), pch=20, xlim=c(-10,max(PI.boot1$lag.upr)), axes=FALSE, xlab="Estimated time between MRCA and diagnosis (years)", ylab="")
segments(x0 = PI.boot1$lag.lwr, x1 = PI.boot1$lag.upr, y0 = 1:nrow(PI.boot1))
axis(side=1, at=(0:5)*10, labels=(0:5)*10)
text(x = PI.boot1$lag.lwr-1, y = 1:nrow(PI.boot1), labels = PI.boot1$LRIID, adj = 1, cex=0.75)
```

### Age of occurrence of t(3;5) events with chr5q gains

To explore the data, we extract the somatically acquired base substitutions in the region of chromosome 5q that is
duplicated. The variant allele fraction for each mutation called in any sample is extracted and then converted to a
cancer cell fraction (CCF; the fraction of cancer cells in the sample that carry the mutation) using the level of
normal cell contamination and copy number at that position. From this, we calculate whether the mutation was acquired
prior to the duplication of chromosome 5q (if it was, the CCF will be close to 2; if not, CCF ~= 1).
For clonal mutations (seen in all samples), we take the consensus across all samples to vote whether it more likely
occurred before or after chr 5q duplication. Results are then plotted for each sample to allow assessment of
consistency of the calls / data. This component is encoded in the function timing() in the code chunk below.

Then, we apply two similar methods to estimate the age of occurrence of the t(3;5) gain:

(1) The first is to use the number of chr 5q mutations that are clonal and acquired before the 5q duplication relative
to the number that are clonal but acquired after duplication (correcting for the fact that post-duplication there is an
extra copy of 5q and hence the mutation burden accumulates more quickly). The fraction of clonal time at which the
duplication occurred is then estimated from the estimate of when the MRCA emerged. Bootstrapping provides 95% confidence
intervals, incorporating the uncertainty in the numbers of pre- and post-duplication mutations and the age the MRCA
emerges (and therefore mutation rate). This estimation is encoded in the function triploid.2.1.with.mrca.est() in the
code chunk below.

(2) The second method is to derive the age of occurrence directly from the mutation rate estimated per patient from
the LME and the number of mutations that have accumulated before the 5q was duplicated. This requires correction for
the size of the region gained and what the total size of the genome that could have had mutations called
(from the BAM files, we estimate that this is 5.32Gb for a typical sample in this series).
Again, bootstrapping provides 95% confidence intervals, incorporating the uncertainty in the numbers of pre-duplication
mutations and the patient-specific mutation rate. This method is encoded in the function triploid.direct.est()
in the code chunk below.

```
# First we define a function that reads in the relevant mutations and decides whether a variant is clonal or subclonal; and if clonal whether it occurs pre- or post-duplication

sa.df <- read.table("../../data/summary/sample_summary.txt", sep="\t", header=TRUE, stringsAsFactors = FALSE)
sa.df <- sa.df[sa.df$include == "yes",]

timing <- function(pt = "PD24236", sample.suffixes = c("a", "c", "d", "e"), normal.contam, chr, lower, upper) 
{
  cn <- list()

  # Read in the mutation data for that patient
  file.name <- system(paste("ls ../../data/patient_data/merged/", pt, "/1152_*_consolidated_snp.tsv", sep=""), intern=TRUE)
  header.line.num <- system(paste("grep -n VariantID ../../data/patient_data/merged/", pt, "/1152_*_consolidated_snp.tsv", sep=""), intern=TRUE)
  header.line.num <- as.double(strsplit(header.line.num[2], split = ":")[[1]][1])
  muts <- read.table(file.name, header=TRUE, blank.lines.skip = TRUE, stringsAsFactors = FALSE, sep="\t", comment.char="#", row.names=NULL, skip=header.line.num-1)
  muts <- muts[muts$Chrom == chr & muts$Pos >= lower & muts$Pos <= upper,]

  # For each sample in that patient, calculate VAF and get CN at each mutated base-pair
  for (j in 1:length(sample.suffixes)) 
  {
    k <- sample.suffixes[j]
    muts[,paste(k,"VAF",sep="_")] <- muts[,paste(pt, k, "_MTR", sep="")] / muts[,paste(pt, k, "_DEP", sep="")]

    # Get CN file for that patient
    cn.file <- system(paste("ls ../../data/sample_data/BB_output/", pt, k, "*subclones.txt", sep=""), intern=TRUE)
    cn <- read.table(cn.file, header=TRUE, sep="\t", stringsAsFactors = FALSE)

    # Get total and minor copy number estimates for each base in the mutations file where the region shows no or minimal (<20%) subclonal CN variation
    muts[, paste(k,"T.Minor.CN",sep="_")]   <- sapply(1:nrow(muts), function(i,x,y) {
            temp.CN <- y$nMin1_A[y$chr == x$Chrom[i] & y$startpos <= x$Pos[i] & y$endpos >= x$Pos[i] & y$frac1_A > 0.8];
            if (length(temp.CN)>0) {return(temp.CN[1])} else {return(NA)}}, 
            x=muts, y=cn)
    muts <- muts[!is.na(muts[,paste(k,"T.Minor.CN",sep="_")]),]
    muts[, paste(k,"T.Total.CN",sep="_")] <- muts[,paste(k,"T.Minor.CN",sep="_")] + sapply(1:nrow(muts), function(i,x,y) {
            temp.CN <- y$nMaj1_A[y$chr == x$Chrom[i] & y$startpos <= x$Pos[i] & y$endpos >= x$Pos[i] & y$frac1_A > 0.8];
            if (length(temp.CN)>0) {return(temp.CN[1])} else {return(NA)}}, 
            x=muts, y=cn)

    # Calculate the CCF (fraction of cancer cells carrying mutation) from VAF and sample-specific normal cell contamination fraction     
    muts[,paste(k,"CCF",sep="_")] <- muts[,paste(k,"VAF",sep="_")] * ((1-normal.contam[j]) * muts[,paste(k,"T.Total.CN",sep="_")] + normal.contam[j] * 2) / (1-normal.contam[j])

    # From CCF, infer whether mutation occurred pre- or post-duplication in that sample
    muts[,paste(k,"Status",sep="_")] <- rep("Uninformative", nrow(muts))
    muts[muts[,paste(k,"T.Total.CN",sep="_")] >= 2 & muts[,paste(k,"T.Minor.CN",sep="_")] == 0 & muts[,paste(k,"CCF",sep="_")] > 1.5, paste(k,"Status",sep="_")] <- "Preduplication"
    muts[muts[,paste(k,"T.Total.CN",sep="_")] >= 2 & muts[,paste(k,"T.Minor.CN",sep="_")] == 0 & muts[,paste(k,"CCF",sep="_")] <= 1.5, paste(k,"Status",sep="_")] <- "Post-duplication"
    muts[muts[,paste(k,"T.Total.CN",sep="_")] == 3 & muts[,paste(k,"T.Minor.CN",sep="_")] == 1 & muts[,paste(k,"CCF",sep="_")] > 1.5, paste(k,"Status",sep="_")] <- "Preduplication"
    muts[muts[,paste(k,"T.Total.CN",sep="_")] == 3 & muts[,paste(k,"T.Minor.CN",sep="_")] == 1 & muts[,paste(k,"CCF",sep="_")] <= 1.5, paste(k,"Status",sep="_")] <- "Post-duplication"
    muts[muts[,paste(k,"T.Total.CN",sep="_")] == 6 & muts[,paste(k,"T.Minor.CN",sep="_")] == 2 & muts[,paste(k,"CCF",sep="_")] > 2.5, paste(k,"Status",sep="_")] <- "Preduplication"
    muts[muts[,paste(k,"T.Total.CN",sep="_")] == 6 & muts[,paste(k,"T.Minor.CN",sep="_")] == 2 & muts[,paste(k,"CCF",sep="_")] <= 2.5, paste(k,"Status",sep="_")] <- "Post-duplication"
    muts[muts[,paste(k,"T.Total.CN",sep="_")] == 5 & muts[,paste(k,"T.Minor.CN",sep="_")] == 2 & muts[,paste(k,"CCF",sep="_")] > 2.5, paste(k,"Status",sep="_")] <- "Preduplication"
    muts[muts[,paste(k,"T.Total.CN",sep="_")] == 5 & muts[,paste(k,"T.Minor.CN",sep="_")] == 2 & muts[,paste(k,"CCF",sep="_")] <= 2.5, paste(k,"Status",sep="_")] <- "Post-duplication"
    muts[muts[,paste(k,"T.Total.CN",sep="_")] >= 4 & muts[,paste(k,"T.Minor.CN",sep="_")] == 1 & muts[,paste(k,"CCF",sep="_")] > 1.5, paste(k,"Status",sep="_")] <- "Preduplication"
    muts[muts[,paste(k,"T.Total.CN",sep="_")] >= 4 & muts[,paste(k,"T.Minor.CN",sep="_")] == 1 & muts[,paste(k,"CCF",sep="_")] <= 1.5, paste(k,"Status",sep="_")] <- "Post-duplication"

  }

  # Now, from all statuses across different samples, get a consensus status
  # First, read in the branch assignments of all mutations
  tree.file <- system(paste("ls params/Tree_structures/", pt, "*optima*", sep=""), intern = TRUE)
  tree.struct <- read.table(file=tree.file, header=TRUE, stringsAsFactors = FALSE, sep="\t")
  branch.assign <- read.table(paste("../../data/patient_data/clusters/", pt, "/", pt, "_assigned_mutations.txt", sep=""), header=TRUE, sep="\t", stringsAsFactors = FALSE)
  branch.assign <- branch.assign[branch.assign$chr == chr & branch.assign$pos >= lower & branch.assign$pos <= upper,]

  muts <- muts[muts$Pos %in% branch.assign$pos,]
  muts$Branch <- sapply(muts$Pos, function(i,x) {x$cluster[x$pos == i]}, x=branch.assign)

  # Identify clonal mutations (assigned to trunk of phylogenetic tree & >1 read reporting variant in all samples)
  muts$Timing <- rep("Subclonal", nrow(muts))
  muts$Timing[muts$Branch %in% (1:nrow(tree.struct))[tree.struct$Name == "T"] &
              sapply(1:nrow(muts), function(i,x,pt.name,samps) {all(x[i,paste(pt.name, samps, "_MTR", sep="")] > 1)}, x=muts, pt.name=pt, samps=sample.suffixes)] <- "Uncertain"
  muts$Timing[sapply(1:nrow(muts), function(i,x) {sum(x[i,grepl("_Status", names(x))] == "Preduplication") > length(sample.suffixes)/2 & x$Timing[i] == "Uncertain"}, x=muts)] <- "Clonal Preduplication"
  muts$Timing[sapply(1:nrow(muts), function(i,x) {sum(x[i,grepl("_Status", names(x))] == "Post-duplication") > length(sample.suffixes)/2 & x$Timing[i] == "Uncertain"}, x=muts)] <- "Clonal Post-duplication"

  # Generate plot of CCF per sample
  for (i in sample.suffixes)
  {
    point.col <- as.character(factor(muts$Timing, levels=c("Clonal Post-duplication", "Clonal Preduplication", "Subclonal", "Uncertain"), labels=c("steelblue2", "springgreen3", "plum3", "black")))
    point.col[muts[,paste(pt, i, "_MTR", sep="")] > 1 & muts$Timing == "Subclonal"] <- "orange"
    plot(muts$Pos, muts[,paste(i, "CCF", sep="_")], pch=20, col=point.col, xlab=paste("Chr", chr, "position"), ylab="Number of mutation copies per cancer cell", las=1, ylim=c(0,max(muts[,paste(i, "CCF", sep="_")])+1))
    legend("topright", pch=20, col=c("steelblue2", "springgreen3", "orange", "plum3", "black"), legend=c("Clonal, post-duplication", "Clonal, pre-duplication", "Subclonal, present in this sample", "Subclonal, absent in this sample", "Uncertain"))
    sanger.sid <- paste(pt, i, sep="")
    lri.sid <- sa.df[sa.df$"sanger.ID"==sanger.sid,2]
    title(main = lri.sid)
  }

  return(muts)
}

  # The first method of calculating timing uses MRCA estimates and fraction of mutations at ploidy 1 and ploidy 2 to estimate length 
 triploid.2.1.with.mrca.est <- function(ploidy.1, ploidy.2, bs, ID, age) 
 {
   # bs is bootstrap output from LME
   point.est <- ploidy.2 / (ploidy.2 + (ploidy.1 - ploidy.2)/3)
   iter <- bs$R
   total.muts <- ploidy.1 + ploidy.2
   prob.2 <- ploidy.2 / total.muts

   resamps <- rbinom(n=iter, size=total.muts, p=prob.2)
   ests.bs <- resamps / (resamps + (total.muts - 2*resamps)/3)
   ests.bs[ests.bs > 1] <- 1

   age.mrca.bs <- bs$t[,ID]
   age.mrca.bs[age.mrca.bs > age] <- age

   ests.bs <- ests.bs * age.mrca.bs

   return(quantile(ests.bs, c(0.5,0.025,0.975)))
 }


 # The second method estimates the timing directly from the estimated mutation rate and number of mutations acquired before duplication
  triploid.direct.est <- function(ploidy.2, bs, ID, dup.region.size.in.Gb, callable.genome.size=5.32) 
 {
   # bs is bootstrap output from above
   iter <- bs$R
   mut.rate.bs <- bs$t[,ID] / callable.genome.size
   resamps <- rpois(n=iter, lambda = ploidy.2)
   ests.bs <- resamps / dup.region.size.in.Gb / mut.rate.bs 

   return(quantile(ests.bs, c(0.5,0.025,0.975)))
 }


 # Set up the data-frame for the results
 tloc.ids = c("PD21872", "PD21874", "PD21877", "PD23583", "PD23585", "PD24231", "PD24232", "PD24234", "PD24235", "PD24236", "PD24237", "PD24239")
 tloc.ids.len <- length(tloc.ids)

 lri.ids <- rep("", tloc.ids.len)
 for (x in 1:tloc.ids.len) {
  lri.ids[x] <- pt.df[pt.df$"sanger.ID"==tloc.ids[x],2]
 }

 t3_5.abs.time <- data.frame(ID = tloc.ids, triploid.lwr = rep(0, tloc.ids.len), triploid.est = rep(0, tloc.ids.len), triploid.upr = rep(0, tloc.ids.len), stringsAsFactors = FALSE)
 row.names(PI.boot1) <- PI.boot1$ID
 t3_5.abs.time$Age <- PI.boot1[t3_5.abs.time$ID, "Age"]
 t3_5.abs.time$MRCA.est <- PI.boot1[t3_5.abs.time$ID, "Age"] - PI.boot1[t3_5.abs.time$ID, "Time.lag"]
 colnames(boot1$t) <- new.dat$Sample
 t3_5.abs.time$Est.mut.rate.per.Gb.per.year <- (fixef(muts.per.year.lmer) + ranef(muts.per.year.lmer)$Sample[t3_5.abs.time$ID,"Age"]) / 6
 t3_5.abs.time$alt.trip.upr <- t3_5.abs.time$alt.trip.est <- t3_5.abs.time$alt.trip.lwr <- rep(0,tloc.ids.len)
 t3_5.abs.time$LRIID <- lri.ids
 row.names(t3_5.abs.time) <- t3_5.abs.time$ID

 # Run the patients through the functions in turn ([params/timing_inputs.csv](file:///params/timing_inputs.csv));
time.inputs <- read.table( "params/timing_inputs.csv", header=F, sep=",", stringsAsFactors=F, strip.white=T, as.is=T )

for (i in 1:nrow(time.inputs)) {
   branch.names <- unlist(strsplit(time.inputs[i,2],";"))
   branch.parms <- as.numeric(unlist(strsplit(time.inputs[i,3],";")))
   branches.pid <- time.inputs[i,1]     # patient ID
   branches.age <- time.inputs[i,8]     # patient age
   timing(branches.pid, branch.names, branch.parms, time.inputs[i,4], time.inputs[i,5], time.inputs[i,6]) -> a
   t3_5.abs.time[t3_5.abs.time$ID == branches.pid, c("triploid.est", "triploid.lwr", "triploid.upr")] <- 
   triploid.2.1.with.mrca.est(ploidy.1 = table(a$Timing)["Clonal Post-duplication"], 
                              ploidy.2 = table(a$Timing)["Clonal Preduplication"], 
                              bs=boot1, ID=branches.pid, age=branches.age)
   t3_5.abs.time[branches.pid, c("alt.trip.est", "alt.trip.lwr", "alt.trip.upr")] <- triploid.direct.est(table(a$Timing)["Clonal Preduplication"], boot2, branches.pid, time.inputs[i,7]/1000)
}
```

```
# Report the results
 # kable(t3_5.abs.time,row.names=FALSE)
 t3_5.abs.time.rep <- t3_5.abs.time
 row.names(t3_5.abs.time.rep) <- t3_5.abs.time$LRIID
 kable(t3_5.abs.time.rep)
```

|  | ID | triploid.lwr | triploid.est | triploid.upr | Age | MRCA.est | Est.mut.rate.per.Gb.per.year | alt.trip.lwr | alt.trip.est | alt.trip.upr | LRIID |
| --- | --- | --- | --- | --- | --- | --- | --- | --- | --- | --- | --- |
| K104 | PD21872 | 10.938395 | 16.432742 | 22.185842 | 58 | 16.43274 | 15.26627 | 11.613700 | 18.527707 | 26.672249 | K104 |
| K108 | PD21874 | 10.739409 | 18.041202 | 26.175018 | 65 | 36.33812 | 14.72333 | 11.522629 | 18.917552 | 27.634223 | K108 |
| K139 | PD21877 | 2.898020 | 7.840651 | 14.074049 | 69 | 44.61501 | 11.94899 | 3.531652 | 8.062710 | 14.330546 | K139 |
| K167 | PD23583 | 5.548462 | 14.556855 | 23.527838 | 49 | 20.60705 | 11.80136 | 2.441123 | 11.249291 | 22.952977 | K167 |
| K65 | PD23585 | 4.067799 | 7.341303 | 11.915922 | 53 | 24.61008 | 10.12517 | 4.218487 | 8.594933 | 14.059000 | K65 |
| K163 | PD24231 | 6.127674 | 11.782681 | 18.789837 | 60 | 39.10981 | 15.60205 | 5.993376 | 10.911055 | 16.302242 | K163 |
| K165 | PD24232 | 2.903123 | 8.840734 | 17.234043 | 54 | 46.78683 | 17.38481 | 2.163165 | 6.561965 | 13.612118 | K165 |
| K176 | PD24234 | 0.000000 | 3.435768 | 9.364139 | 60 | 57.69398 | 15.02653 | 0.000000 | 2.916591 | 7.814361 | K176 |
| K180 | PD24235 | 11.133159 | 19.945748 | 30.953686 | 78 | 41.95394 | 13.48045 | 12.470850 | 20.426234 | 30.978560 | K180 |
| K96 | PD24236 | 16.635997 | 26.046465 | 34.535433 | 68 | 64.81986 | 15.35655 | 18.796737 | 27.920701 | 38.766939 | K96 |
| K156 | PD24237 | 9.708996 | 16.910246 | 25.641266 | 70 | 43.98573 | 12.82140 | 11.242602 | 19.223540 | 28.502538 | K156 |
| K23 | PD24239 | 9.598668 | 17.564885 | 25.652174 | 59 | 55.22055 | 16.50226 | 8.289134 | 16.062970 | 25.860163 | K23 |

```
 # Generate plot of estimated age of occurrence of t(3;5) - method 1
 t3_5.abs.time <- t3_5.abs.time[order(t3_5.abs.time$triploid.est),]
 plot(t3_5.abs.time$triploid.est, 1:nrow(t3_5.abs.time), pch=20, xlim=c(0,max(t3_5.abs.time$triploid.upr)), axes=FALSE, xlab="Estimated age at t(3;5) (years)", ylab="")
 segments(x0 = t3_5.abs.time$triploid.lwr, x1 = t3_5.abs.time$triploid.upr, y0 = 1:nrow(t3_5.abs.time))
 axis(side=1, at=(0:7)*5, labels=(0:7)*5)
 text.x.poss <- t3_5.abs.time$triploid.lwr-0.5
 text.x.poss[1] <- text.x.poss[1] + 0.5
 text(x = text.x.poss, y = 1:nrow(t3_5.abs.time), labels = t3_5.abs.time$LRIID, adj = 1, cex=0.75)
 title("Estimated age of t(3;5) - method 1")
```

```
  # Generate plot of estimated age of occurrence of t(3;5) - method 2
 plot(t3_5.abs.time$alt.trip.est, 1:nrow(t3_5.abs.time), pch=20, xlim=c(0,max(t3_5.abs.time$alt.trip.upr)), axes=FALSE, xlab="Estimated age at t(3;5) (years)", ylab="")
 segments(x0 = t3_5.abs.time$alt.trip.lwr, x1 = t3_5.abs.time$alt.trip.upr, y0 = 1:nrow(t3_5.abs.time))
 axis(side=1, at=(0:7)*5, labels=(0:7)*5)
 text.x.poss <- t3_5.abs.time$alt.trip.lwr-0.5
 text.x.poss[1] <- text.x.poss[1] + 0.5
 text(x = text.x.poss, y = 1:nrow(t3_5.abs.time), labels = t3_5.abs.time$LRIID, adj = 1, cex=0.75)
 title("Estimated age of t(3;5) - method 2")
```

## Rate of VHL driver mutations

To estimate the average rate of driver mutations in VHL, we estimate separately the rate of substitutions and indels. To estimate the rate of driver substitutions, we calculate the estimated rate of each of the 6 mutation types in each of the trinucleotide contexts from the overall substitution rate and the observed sequence context of kidney cancer point mutations. We then take the length and sequence composition of the CDS of VHL and generate all possible substitutions, and from this extract the set of all possible amino acid consequences arising from substitutions along the length of VHL. From this, we define the set of all possible driver substitutions as any substitutions that are: start-lost, stop-lost, stop-gained or a member of the set of previously observed amino acid substitutions in VHL recorded in clear cell RCCs in the COSMIC database. The rates of these individual mutations are then summed to generate the overall driver substitution rate per cell per year.

```
rev.comp <- function(x) {
  x <- toupper(x)
  x <- rev(unlist(strsplit(x,"")))
  x <- paste(sapply(x, switch,  "A"="T", "T"="A","G"="C","C"="G"),collapse="")
  return(x)
}

# Define the per year per genome substitution rate
rate.lmer <- lmer(Num.mutations ~ Age - 1 + (Age - 1 | Sample), data=branch.df)
SUB.RATE <- fixef(rate.lmer)["Age"]
CALLABLE.GENOME.SIZE <- 5.32E9

# Read in the data for the gene and mutation signatures
# gene.cds has to include 1 non-coding base before CDS and one after for mutation signature analysis
gene.cds <- substring(read.table(file="../../data/sampler_data/VHL_transcript.txt", header=FALSE, stringsAsFactors = FALSE, fill=TRUE)$V1[1], 213, 856)
cosmic.muts <- read.csv("../../data/sampler_data/COSMIC_VHL_systematic_somatic.csv", quote="", stringsAsFactors = FALSE)

# Read in and manipulate the file for random positions in the genome
rand.pos <- read.table("../../data/sampler_data/Random_build37_positions_with_context.txt", header=TRUE, sep="\t", stringsAsFactors = FALSE)
rand.pos <- rand.pos[!grepl("N", rand.pos$CONTEXT, fixed=TRUE),]
rand.pos$Pyrimidine.WT <- rand.pos$WT
rand.pos$Pyrimidine.WT[rand.pos$WT == "A"] <- "T"
rand.pos$Pyrimidine.WT[rand.pos$WT == "G"] <- "C"
rand.pos$Pyrimidine.context <- substring(rand.pos$CONTEXT, 10, 12)
rand.pos$Pyrimidine.context[rand.pos$WT %in% c("A", "G")] <- sapply(rand.pos$Pyrimidine.context[rand.pos$WT %in% c("A", "G")], rev.comp)

# Now, calculate the per trinucleotide per mutation type mutation rate
observed.mut.spectrum <- read.table("../../data/sampler_data/t3_5_pts_chr5_muts.txt", header=TRUE, sep="\t", stringsAsFactors = FALSE)
observed.mut.spectrum$Pyrimidine.WT <- observed.mut.spectrum$WT
observed.mut.spectrum$Pyrimidine.WT[observed.mut.spectrum$WT == "A"] <- "T"
observed.mut.spectrum$Pyrimidine.WT[observed.mut.spectrum$WT == "G"] <- "C"
observed.mut.spectrum$Pyrimidine.Alt <- observed.mut.spectrum$Alt
observed.mut.spectrum$Pyrimidine.Alt[observed.mut.spectrum$WT %in% c("A", "G")] <- sapply(observed.mut.spectrum$Pyrimidine.Alt[observed.mut.spectrum$WT %in% c("A", "G")], rev.comp)
observed.mut.spectrum$Pyrimidine.context <- substring(observed.mut.spectrum$CONTEXT, 10, 12)
observed.mut.spectrum$Pyrimidine.context[observed.mut.spectrum$WT %in% c("A", "G")] <- sapply(observed.mut.spectrum$Pyrimidine.context[observed.mut.spectrum$WT %in% c("A", "G")], rev.comp)
observed.mut.spectrum$Mut.type <- paste(observed.mut.spectrum$Pyrimidine.WT, ">", observed.mut.spectrum$Pyrimidine.Alt, sep="")
obs.mut.table <- table(observed.mut.spectrum$Mut.type, observed.mut.spectrum$Pyrimidine.context) / nrow(observed.mut.spectrum)

trinucs.in.genome <- table(rand.pos$Pyrimidine.context) / nrow(rand.pos)
mut.rate.per.type.per.trinuc <- data.frame(Mut.type = rep(sort(unique(observed.mut.spectrum$Mut.type)), each=16), 
                                           Trinuc = c(rep(paste(rep(c("A", "C", "G", "T"), each=4), "C", rep(c("A", "C", "G", "T"), 4), sep=""), 3), rep(paste(rep(c("A", "C", "G", "T"), each=4), "T", rep(c("A", "C", "G", "T"), 4), sep=""), 3)),
                                           stringsAsFactors = FALSE)
Prob.per.mut.type.per.trinuc <- sapply(1:nrow(mut.rate.per.type.per.trinuc), function(i,x,y,z) {y[x$Mut.type[i], x$Trinuc[i]] / (z[x$Trinuc[i]] * CALLABLE.GENOME.SIZE * 2)}, x=mut.rate.per.type.per.trinuc, y=obs.mut.table, z=trinucs.in.genome) # Need to double callable genome size because diploid and looking for VHL mutation on specific parental haplotype
names(Prob.per.mut.type.per.trinuc) <- paste(mut.rate.per.type.per.trinuc$Mut.type, mut.rate.per.type.per.trinuc$Trinuc, sep="::")

# Read in the genetic code
genetic.code.df <- read.table("../../data/sampler_data/Genetic_code.txt", sep=" ", stringsAsFactors = FALSE, col.names=c("codon", "aa", "aa.code"))
genetic.code <- genetic.code.df$aa.code
names(genetic.code) <- genetic.code.df$codon
genetic.code <- substring(genetic.code, 2, 2)

# Generate all possible mutations
poss.drivers <- data.frame(Ref = rep(unlist(strsplit(substring(gene.cds,2,nchar(gene.cds)-1), split = "")), each=4), 
                           Alt = rep(c("A", "C", "G", "T"), nchar(gene.cds)-2), 
                           cDNA.pos = rep(1:(nchar(gene.cds)-2), each=4),
                           aa.pos = rep(1:((nchar(gene.cds)-2)/3), each=12),
                           Pos.in.codon = rep(rep(1:3, each=4), (nchar(gene.cds)-2)/3),
                           Base.5prime = rep(unlist(strsplit(substring(gene.cds,1,nchar(gene.cds)-2), split = "")), each=4),
                           Base.3prime = rep(unlist(strsplit(substring(gene.cds,3,nchar(gene.cds)), split = "")), each=4),
                           Codon = rep(sapply(seq(2,nchar(gene.cds)-3,3), function(i,x) substring(x, i, i+2), x=gene.cds), each=12), stringsAsFactors = FALSE)

poss.drivers$Alt.codon <- sapply(1:nrow(poss.drivers), function(i,x) {cod <- unlist(strsplit(x$Codon[i],"")); cod[x$Pos.in.codon[i]] <- x$Alt[i]; return(paste(cod, collapse=""))}, x=poss.drivers)
poss.drivers <- poss.drivers[genetic.code[poss.drivers$Codon] != genetic.code[poss.drivers$Alt.codon],]
poss.drivers$Mut.string <- paste("p.", genetic.code[poss.drivers$Codon], poss.drivers$aa.pos, genetic.code[poss.drivers$Alt.codon], sep="")

poss.drivers$Pyrimidine.WT <- poss.drivers$Ref
poss.drivers$Pyrimidine.WT[poss.drivers$Ref == "A"] <- "T"
poss.drivers$Pyrimidine.WT[poss.drivers$Ref == "G"] <- "C"
poss.drivers$Pyrimidine.Alt <- poss.drivers$Alt
poss.drivers$Pyrimidine.Alt[poss.drivers$Ref %in% c("A", "G")] <- sapply(poss.drivers$Pyrimidine.Alt[poss.drivers$Ref %in% c("A", "G")], rev.comp)
poss.drivers$Pyrimidine.context <- paste(poss.drivers$Base.5prime, poss.drivers$Ref, poss.drivers$Base.3prime, sep="")
poss.drivers$Pyrimidine.context[poss.drivers$Ref %in% c("A", "G")] <- sapply(poss.drivers$Pyrimidine.context[poss.drivers$Ref %in% c("A", "G")], rev.comp)
poss.drivers$Mut.type <- paste(poss.drivers$Pyrimidine.WT, ">", poss.drivers$Pyrimidine.Alt, sep="")

poss.drivers <- poss.drivers[poss.drivers$aa.pos == 1 |
  (genetic.code[poss.drivers$Alt.codon] == "X" & genetic.code[poss.drivers$Codon] != "X") |
  (genetic.code[poss.drivers$Alt.codon] != "X" & genetic.code[poss.drivers$Codon] == "X") |
  poss.drivers$Mut.string %in% cosmic.muts$AA.Mutation,]

# Calculate estimated VHL driver substitution rate per year
vhl.driver.sub.rate <- sum(Prob.per.mut.type.per.trinuc[paste(poss.drivers$Mut.type, poss.drivers$Pyrimidine.context, sep="::")]) * SUB.RATE

print(paste("Average driver substitution rate in VHL:", signif(vhl.driver.sub.rate, digits = 2), "/cell/year"))
```

[1] “Average driver substitution rate in VHL: 8.5e-07 /cell/year”

Estimating the rate of driver indels in VHL follows broadly the same approach. We show that there is a strongly linear association between the number of substitutions and the number of indels across patients in the cohort. Using the slope of this relationship, we estimate the indel rate per year per clone from the average substitution rate. We then assume that all indels within the CDS of VHL are driver mutations. This then allows us to calculate the VHL driver indel mutation rate per cell per year.

```
# Now we need to consider indel drivers
# The indel rate is ~5% the SUB.RATE, and we can consider all indels occurring within VHL CDS as drivers
vhl.driver.indel.rate <- (1/8.5) * SUB.RATE / CALLABLE.GENOME.SIZE * 644
print(paste("Average driver indel rate in VHL:", signif(vhl.driver.indel.rate, digits = 2), "/cell/year"))
```

[1] “Average driver indel rate in VHL: 1.2e-06 /cell/year”

```
# Therefore
vhl.driver.mutation.rate <- vhl.driver.indel.rate + vhl.driver.sub.rate
print(paste("Average driver point mutation rate in VHL:", signif(vhl.driver.mutation.rate, digits = 2), "/cell/year"))
```

[1] “Average driver point mutation rate in VHL: 2.1e-06 /cell/year”

## Models of age-incidence curves for sporadic & inherited ccRCC

There is a rich literature of using age-incidence curves for cancer to derive fundamental insights into the dynamics of cancer evolution. What we have learnt in this study is that the evolution of sporadic clear cell kidney cancer appears to follow well-defined and recurrent trajectories. Frequently, it seems the first event is chromosome 3p loss, often with concomitant gains on other chromosomes - this appeors to occur predominantly in childhood or adolescence. The other key event that occurs early in the evolution of sporadic ccRCC is inactivation of the other allele of VHL, typically through point mutation (notwithstanding the role of epigenetic silencing) - this is an obligately early event, because it is both highly recurrent across patients (>75%) and always present on the trunk of the phylogenetic tree.

There are clearly other key steps in the evolution of ccRCC. These include: modulation of the PI3K signalling pathway; TERT up-regulation (often through regulatory mutations); driver mutations in other epigenetic regulators such as PBRM1 and/or SETD2; other structural variations and copy number changes; and clonal expansion of the most recent common ancestor. These other driver mutations typically occur later than chr 3p loss and VHL inactivation (since they are often subclonal) and each occurs with a recurrence rate that is considerably lower (typically <20% patients).

Furthermore, exploring the genomic features of ccRCC that have occurred in the setting of inherited VHL mutations reveals many similarities with sporadic ccRCC, as described by Fei et al and analysed further here. Large-scale chromosomal loss of the other allele of chr 3p is universal. The other driver mutations occur in the same genes and at broadly the same frequencies. The mutation rate is similar to that seen in sporadic cancers, with an almost identical spectrum and linear association with age.

We therefore built Bayesian models of the age-incidence curves for sporadic and inherited ccRCC. The first step is to interpret the published age-incidence figures. The age-incidence curves for sporadic clear cell RCC come from Cancer Research UK and represent age-specific annual incidence figures per 100,000 population, banded in 5-year groups. The figures for inherited vHL disease are Kaplan-Meier curves derived from Ong et al. The second step is to take draws of cohorts of individual patients (or censored non-patients) from the published curves. These then represent the data that is fitted by the Bayesian model.

```
NUM.VHL.CASES <- 100
NUM.SPORADIC <- 1000

# Read in age-incidence curves
vhl.penetrance <- read.table("../../data/sampler_data/Penetrance of RCC in vHL disease.txt", sep="\t", header=TRUE, stringsAsFactors = FALSE)
cruk.sporadic.incidence <- read.table("../../data/sampler_data/Age-incidence of sporadic RCC.txt", sep="\t", header=TRUE, stringsAsFactors = FALSE)

# Generate random sample of NUM.VHL.CASES age-incidences and censoring values for inherited RCCs
# Note that the vhl.penetrance data are cumulative Kaplan-Meier incidences and occur in nearly yearly increments
vhl.penetrance <- rbind(vhl.penetrance, c(63,1))
vhl.penetrance$lower <- c(0, vhl.penetrance$Penetrance[1:(nrow(vhl.penetrance)-1)])

vhl.incidence <- data.frame(Type = rep("vHL", NUM.VHL.CASES), stringsAsFactors = FALSE)
draws <- runif(n = NUM.VHL.CASES, min=0, max=1)
vhl.incidence$Age <- sapply(draws, function(i,x) x$Age[x$lower <= i & x$Penetrance > i], x=vhl.penetrance) 
vhl.incidence$Censored <- as.double(vhl.penetrance$lower[nrow(vhl.penetrance)] <= draws)

# Generate random sample of NUM.CASES age-incidences and censoring values for sporadic RCCs
# Note that the cruk.sporadic.incidence data are age-incidence rates per year per 100,000 population in 5 year blocks
cruk.sporadic.incidence$Rate.per.year <- (cruk.sporadic.incidence$Male.Rates + cruk.sporadic.incidence$Female.Rates)/2/100000
cruk.by.year <- data.frame(Age = 1:95, 
                           Incidence = rep(cruk.sporadic.incidence$Rate.per.year, each=5))
cruk.by.year$Penetrance <- 1 - cumprod(1-cruk.by.year$Incidence)
cruk.by.year <- rbind(cruk.by.year, c(95, 1, 1))
cruk.by.year$lower <- c(0, cruk.by.year$Penetrance[1:(nrow(cruk.by.year)-1)])


sporadic.incidence <- data.frame(Type = rep("Sporadic", NUM.SPORADIC), stringsAsFactors = FALSE)
draws <- runif(n = NUM.SPORADIC, min=0, max=1)
sporadic.incidence$Age <- sapply(draws, function(i,x) x$Age[x$lower <= i & x$Penetrance > i], x=cruk.by.year) 
sporadic.incidence$Censored <- as.double(cruk.by.year$lower[nrow(cruk.by.year)] <= draws)
```

### Bayesian model of age-incidence curves

The primary question of interest is how large is the clone(s) carrying 3p loss during the development of sporadic kidney cancer. The intuition here is a twist on the approach Knudson took in deriving his two-hit model of retinoblastoma incidence. Essentially, he takes the known number of retinal ganglion cells and age-incidence of inherited retinoblastomas to derive the mutation rate in the (then unknown) predisposition gene. He then shows that the age-incidence curves for sporadic retinoblastoma fit with a two-mutation model using the estimated mutation rate. In our situation, we know the mutation rate (and target gene) very accurately - indeed, in the preceding section, we estimate the *VHL* driver mutation rate per clone per year. From the difference in age-incidence curves between *VHL* carriers and sporadic kidney cancers, and the known *VHL* driver mutation rate per cell per year, we can estimate how many cells that are susceptible to initiating ccRCC carry chr 3p loss during adulthood.

The major assumption in this approach is that the evolution of sporadic kidney cancers and inherited ccRCC in vHL patients is identical except for the need to inactivate *VHL* as a somatic event in the sporadic cases. The broad concept for modelling the age-incidence curves is to treat sporadic ccRCC as the sum of three independent waiting times (time to 3p loss; time from 3p loss to *VHL* inactivation; time from biallelic *VHL* loss to diagnosed kidney cancer). We treat ccRCC in carriers of VHL as the sum of two independent waiting times, with the same distribution as in sporadic cases (time to 3p loss; time from 3p loss (that is, biallelic *VHL* inactivation) to diagnosed kidney cancer).

In more technical detail, we let \(Y\_{i,spor}\) denote the age of incidence (in years) of patient \(i\) with sporadic ccRCC, and \(Y\_{i,vHL}\) the age of incidence of ccRCC in patients with von Hippel-Lindau disease, we have:
\[
\begin{align}
Y\_{i,spor} &= Z\_1 + Z\_2 + Z\_3\
Y\_{i,vHL} &= Z\_1 + Z\_3,
\end{align}
\]
where \(Z\_1\) is the time to 3p loss; \(Z\_2\) is the time to *VHL* inactivation; and \(Z\_3\) is the time from (biallelic) *VHL* loss to kidney cancer diagnosis. Clearly, we do not observe all waiting times, since ~20% of vHL cases do not develop ccRCC and the vast majority on non-carriers do not. Thus, there will be censoring of the sum of waiting times for many individuals, which we handle by data augmentation.

We model these waiting times with the gamma distribution for \(Z\_1\) and \(Z\_3\) and the exponential distribution for \(Z\_2\). That is:
\[
\begin{align}
Z\_1 &\sim \Gamma(\alpha\_1, \beta\_1)\
Z\_2 &\sim \text{Exp}(\lambda)\
Z\_3 &\sim \Gamma(\alpha\_3, \beta\_3),
\end{align}
\]
where \(\lambda = n\mu\), with \(n\) as the number of cells in the clone after chr 3p loss and \(\mu\) as the *VHL* driver mutation rate per cell per year. We use the conjugate prior: \(\lambda \sim \Gamma(0.01,0.01)\). The parameters, \(\alpha\_1, \beta\_1, \alpha\_3, \beta\_3\), we model as coming from the conjugate prior to the gamma distribution:
\[(\alpha, \beta) \propto \frac{p^{\alpha-1}e^{-\beta q}}{\Gamma(\alpha)^r\beta^{-\alpha s}},\]
where p, q, r and s are the hyperparameters of the conjugate prior. For \(\alpha\_3\) and \(\beta\_3\), we use uninformative hyperparameters (\(p\_3=q\_3=r\_3=s\_3=1\)), but for \(\alpha\_1\) and \(\beta\_1\), we put an informative prior distilled from the estimated ages of chromosome 3p loss. That is,

\[p\_1 = \prod\_i{x\_{3p,i}}~;~~q\_1 = \sum\_i{x\_{3p,i}}~;~~ r\_1 = s\_1 = n\_{3p}~,\]

where \(x\_{3p,i}, i=1,…,n\_{3p}\) are the estimated ages at which chr 3p loss occurred from patients with informative t(3;-) translocations.

We take draws from the posterior distribution using a Gibbs sampler. We use 50,000 iterations with the first 20,000 being treated as burn-in. The steps involved are as follows:

1. **Update \(z\_{i,1}\) and \(z\_{i,3}\) for vHL patients who were not censored.** Since patients who were not censored have an exact observed age of incidence, \(y\_i\), we must take draws such that \(z\_{i,1} + z\_{i,3} = y\_i\). We do this by using a Metropolis-Hastings approach where the proposal distribution is a Dirichlet distribution, scaled to the age of incidence. That is,
   \[(z\_{i,1}^{(\*)},z\_{i,3}^{(\*)}) \sim y\_i~.~\text{Dir}\left(\frac{\kappa~z\_{i,1}^{(j-1)}}{z\_{i,1}^{(j-1)}+z\_{i,3}^{(j-1)}},~ \frac{\kappa~z\_{i,3}^{(j-1)}}{z\_{i,1}^{(j-1)}+z\_{i,3}^{(j-1)}}\right),\]
   where \(\kappa\) is a scaling factor tuned by the user to optimise the acceptance ratio of the M-H algorithm. The importance ratio for the M-H algorithm is therefore:
   \[
   \begin{align}
   Q\_i &= \frac{f({\bf{z\_i^{(j-1)}}}|{\bf{z\_i^{(\*)}}})}{f({\bf{z\_i^{(\*)}}}|{\bf{z\_i^{(j-1)}}})}.\frac{P({\bf{z\_i^{(\*)}}}|{\bf{z\_i^{(j-1)}}}, \alpha\_1^{(j-1)}, \beta\_1^{(j-1)}, \alpha\_3^{(j-1)}, \beta\_3^{(j-1)}))}{P({\bf{z\_i^{(j-1)}}}|{\bf{z\_i^{(\*)}}}, \alpha\_1^{(j-1)}, \beta\_1^{(j-1)}, \alpha\_3^{(j-1)}, \beta\_3^{(j-1)})}\
   & \
   &= \frac{f({\bf{z\_i^{(j-1)}}}|{\bf{z\_i^{(\*)}}})}{f({\bf{z\_i^{(\*)}}}|{\bf{z\_i^{(j-1)}}})}.
   \left( \frac{z\_{i,1}^{(\*)}}{z\_{i,1}^{(j-1)}} \right)^{\alpha\_1^{(j-1)}-1}.
   \left( \frac{z\_{i,3}^{(\*)}}{z\_{i,3}^{(j-1)}} \right)^{\alpha\_3^{(j-1)}-1}.
   e^{-\beta\_1^{(j-1)}(z\_{i,1}^{(\*)}-z\_{i,1}^{(j-1)})-\beta\_3^{(j-1)}(z\_{i,3}^{(\*)}-z\_{i,3}^{(j-1)})}
   \end{align}
   \]
   This is the accepted or reject in the usual way.
2. **Update of \(z\_{i,1}\) and \(z\_{i,3}\) for vHL patients who were censored.** Here, we know only the lower bound on the \(y\_i\), so we sample these using rejection sampling. That is, we take draws of \(z\_{i,1}^{(\*)} \sim \Gamma \left(\alpha\_1^{(j-1)}, \beta\_1^{(j-1)} \right)\) and \(z\_{i,3}^{(\*)} \sim \Gamma \left(\alpha\_3^{(j-1)}, \beta\_3^{(j-1)} \right)\) until \(z\_{i,1}^{(\*)} + z\_{i,3}^{(\*)}\) is greater than the age of censoring.
3. **Update of \(z\_{i,1}\), \(z\_{i,2}\) and \(z\_{i,3}\) for sporadic ccRCC patients who were not censored.** We apply the same approach as in step 1, using an analogous three parameter Dirichlet proposal distribution.
4. **Update of \(z\_{i,1}\), \(z\_{i,2}\) and \(z\_{i,3}\) for sporadic ccRCC patients who were censored.** We apply the analogous rejection sampling approach as used in step 2.
5. **Update of \(\alpha\_1\), \(\beta\_1\), \(\alpha\_3\) and \(\beta\_3\) from cojugate prior.** Given the (informative) hyperparameters \(p\_1, q\_1, r\_1\text{ and }s\_1\) for the conjugate prior for \(\alpha\_1\) and \(\beta\_1\), we have:
   \[f(\alpha\_1, \beta\_1 | p\_1, q\_1, r\_1, s\_1,{\bf{z\_1}}) \propto \frac{\left(p\_1\prod\_iz\_i \right)^{\alpha\_1}~.~e^{-\beta\_1(q\_1+\sum\_iz\_i)}}{\Gamma(\alpha\_1)^{r\_1+n}~.~\beta\_1^{-\alpha\_1(s\_1+n)}}\]
   We sample from this posterior using Metropolis-Hastings with the proposal distributions as independent gamma variables: \(\alpha\_1^{(\*)} \sim \Gamma \left(\gamma \alpha\_1^{(j-1)}, \gamma \right),~~\beta\_i^{(\*)} \sim \Gamma \left(\gamma \beta\_1^{(j-1)},\gamma \right),\) where \(\gamma\) is a scaling factor defined by the user to optimise the acceptance / rejection ratio. The importance ratio is calculated, and the proposed values, \(\alpha\_1^{(\*)}\) and \(\beta\_1^{(\*)}\), acceoted or rejected in the usual way. The same approach is applied to updating \(\alpha\_3\) and \(\beta\_3\).
6. **Update of \(\lambda\).** We take draws directly from the posterior: \(\lambda^{(j)} \sim \Gamma \left( 0.01+n\_{spor}, 0.01+\sum\_i z\_{i,2}^{(j)} \right)\).
7. **Draws from posterior distribution of ages of incidence under different clone sizes.** We can also take draws of alternative \(z\_{i,2}\) values if the clone size were different. That is, we draw \(z\_{i,2}^{(j)} \sim \text{Exp} (\rho \lambda^{(j)})\) and add to \(z\_{i,1}^{(j)}+z\_{i,3}\){(j)}$ to generate an alternative age of incidence.

There are a few points of note in this implementation of the model. First, we assume independence of the waiting times. If, for example, there is clone-to-clone variation in the mutation rate, this assumption may not be entirely valid, since \(Z\_2\) would potentially be correlated with \(Z\_3\). However, it is unclear how much clone-to-clone variation there is in mutation rate among kidney cells and how much inter-individual variation. Second, we assume that the clone size is broadly constant after chromosome 3p loss. That is, the clone expands rapidly to a steady-state number, at which stage it plateaus. In fact, given the relatively small number of cells estimated in the clones, this is probably a reasonable assumption. Third, we do make the assumption that the order of events is chromosome 3p loss, followed by *VHL* point mutation, followed by other driver mutations, clonal expansion and diagnosis. Given that we are estimating a clone size of several hundred cells after chromosome 3p loss, it is statistically much more likely that the *VHL* mutation will occur after this clonal expansion than in the one cell before chr 3p loss (inherent in this is the assumption that the rate of chromosome 3p loss through, for example, chromothripsis involving chromosomes 3p and 5q is much lower than that of *VHL* driver point mutations).

```
# Generate MLE estimators for gamma distribution of age of 3p loss
ages.3p.loss <- t3_5.abs.time$triploid.est
s <- log(mean(ages.3p.loss)) - mean(log(ages.3p.loss))
alpha.1.est <- (3 - s + sqrt((s-3)^2 + 24*s)) / (12*s)
beta.1.est <- alpha.1.est / mean(ages.3p.loss)

alpha.1.est
```

[1] 4.231739

```
beta.1.est
```

[1] 0.3009428

```
# Set-up the Gibbs sampler
rcc.age.gs <- function(spor.ages, vhl.ages, chr3p.ages, d.sf, g.sf, iterations, alpha.1.init = alpha.1.est, beta.1.init = beta.1.est, lambda.init=1e-3) {
  # Gibbs sampler for the three waiting times model of RCC development
  # spor.ages is a data.frame of ages of incidence / censoring of sporadic RCC
  # vhl.ages is a data.frame of ages of incidence / censoring of RCC in vHL disease
  # chr3p.ages is a vector of ages of incidence of chr3p loss inferred from t(3;-) cases
  # d.sf is the scaling factor for the Dirichlet proposal distribution - larger values mean smaller jumps
  # g.sf is the scaling factor for the Gamma proposal distribution - larger values mean smaller jumps
  # iterations is the number of iterations of the Gibbs sampler

  # Split age-incidences into censored and non-censored
  spor.ages.cens.T <- spor.ages[spor.ages$Censored == 1,]
  spor.ages.cens.F <- spor.ages[spor.ages$Censored == 0,]
  vhl.ages.cens.T <- vhl.ages[vhl.ages$Censored == 1,]
  vhl.ages.cens.F <- vhl.ages[vhl.ages$Censored == 0,]

  age.of.censoring.vhl <- unique(vhl.ages.cens.T$Age)
  age.of.censoring.sporadic <- unique(spor.ages.cens.T$Age)
  if (length(c(age.of.censoring.vhl,age.of.censoring.sporadic)) != 2) {stop("Age at censoring should be unique")} 

  # Set up initial values and hyperparameters
  alpha.1 <- beta.1 <- alpha.3 <- beta.3 <- accepted.1 <- accepted.3 <- lambda <- rep(NA, iterations)
  alpha.1[1] <- alpha.1.init
  beta.1[1] <- beta.1.init

  alpha.3[1] <- 20
  beta.3[1] <- 0.5

  accepted.1[1] <- accepted.3[1] <- TRUE # Acceptance rates for the M-H algorithm for the Z1 & Z3 gamma parameters

  lambda[1] <- lambda.init # The VHL driver mutation rate per year

  # Values for hyperparameters on gamma distribution for Z1
  log.hyperparameter.p.1 <- sum(log(chr3p.ages))
  hyperparameter.q.1 <- sum(chr3p.ages)
  hyperparameter.r.1 <- hyperparameter.s.1 <- length(chr3p.ages)

  # Set up matrices of waiting times
  vhl.wait.times.cens.F <- array(data = NA, dim=c(nrow(vhl.ages.cens.F), 2, iterations))
  vhl.wait.times.cens.F[,1,1] <- rep(15, nrow(vhl.ages.cens.F))
  vhl.wait.times.cens.F[,2,1] <- vhl.ages.cens.F$Age - 15

  vhl.wait.times.cens.T <- array(data = NA, dim=c(nrow(vhl.ages.cens.T), 2, iterations))
  vhl.wait.times.cens.T[,1,1] <- rep(15, nrow(vhl.ages.cens.T))
  vhl.wait.times.cens.T[,2,1] <- vhl.ages.cens.T$Age - 5

  spor.wait.times.cens.F <- array(data = NA, dim=c(nrow(spor.ages.cens.F), 3, iterations))
  spor.wait.times.cens.F[,1,1] <- rep(15, nrow(spor.ages.cens.F))
  spor.wait.times.cens.F[,2,1] <- (spor.ages.cens.F$Age - 15) / 2
  spor.wait.times.cens.F[,3,1] <- (spor.ages.cens.F$Age - 15) / 2

  spor.wait.times.cens.T <- array(data = NA, dim=c(nrow(spor.ages.cens.T), 3, iterations))
  spor.wait.times.cens.T[,1,1] <- rep(15, nrow(spor.ages.cens.T))
  spor.wait.times.cens.T[,2,1] <- (spor.ages.cens.T$Age - 5) / 2
  spor.wait.times.cens.T[,3,1] <- (spor.ages.cens.T$Age - 5) / 2

  sampled.wait.times.less0 <- sampled.wait.times.less0.25 <- sampled.wait.times.less0.5 <- sampled.wait.times.less0.75 <- matrix(NA, nrow=nrow(spor.ages), ncol=iterations)

  # Run the Gibbs sampler
  for (iter in 2:iterations) {

    ########################################################## 
    # Update of Z1 and Z3 for VHL cases that were not censored
    # Done by Metropolis-Hastings with proposal distribution as Dirichlet centred on curr Z1 & Z3 and scaled
    # Choice of Dirichlet enforces constraint that waiting times must sum to the age of incidence (with scaling)
    vhl.wait.times.cens.F[,,iter] <- censored.wait.times.GS(vhl.wait.times.cens.F[,,iter-1], d.sf, c(alpha.1[iter-1], alpha.3[iter-1]), c(beta.1[iter-1], beta.3[iter-1]))

    ######################################################
    # Update of Z1 and Z3 for vHL cases that were censored
    # Done with rejection sampling
    counter <- 1
    while(counter <= nrow(vhl.ages.cens.T)) {
      z1.proposed <- rgamma(1, alpha.1[iter-1], beta.1[iter-1])
      z3.proposed <- rgamma(1, alpha.3[iter-1], beta.3[iter-1])
      age.proposed <- z1.proposed + z3.proposed

      if (age.proposed > age.of.censoring.vhl) {
        vhl.wait.times.cens.T[counter,1,iter] <- z1.proposed
        vhl.wait.times.cens.T[counter,2,iter] <- z3.proposed
        counter <- counter+1
      } 
    }

    ###################################################################
    # Update of Z1, Z2 and Z3 for sporadic cases that were not censored
    # Done by Metropolis-Hastings with proposal distribution as Dirichlet centred on curr Z1, Z2 & Z3 and scaled
    # Choice of Dirichlet enforces constraint that waiting times must sum to the age of incidence (with scaling)
    spor.wait.times.cens.F[,,iter] <- censored.wait.times.GS(spor.wait.times.cens.F[,,iter-1], d.sf, c(alpha.1[iter-1], 1, alpha.3[iter-1]), c(beta.1[iter-1], lambda[iter-1], beta.3[iter-1]))

    ##########################################################
    # Update of Z1, Z2 and Z3 for vHL cases that were censored
    # Done with rejection sampling
    counter <- 1
    while(counter <= nrow(spor.ages.cens.T)) {
      z1.proposed <- rgamma(1, alpha.1[iter-1], beta.1[iter-1])
      z2.proposed <- rexp(1, lambda[iter-1])
      z3.proposed <- rgamma(1, alpha.3[iter-1], beta.3[iter-1])
      age.proposed <- z1.proposed + z2.proposed + z3.proposed

      if (age.proposed > age.of.censoring.sporadic) {
        spor.wait.times.cens.T[counter,1,iter] <- z1.proposed
        spor.wait.times.cens.T[counter,2,iter] <- z2.proposed
        spor.wait.times.cens.T[counter,3,iter] <- z3.proposed
        counter <- counter+1
      } 
    }

    ##############################################
    # Update age-incidence distribution parameters
    update.1 <- gamma.parameter.GS(alpha.1[iter-1], beta.1[iter-1], log.hyperparameter.p.1, hyperparameter.q.1, hyperparameter.r.1, hyperparameter.s.1, c(vhl.wait.times.cens.T[,1,iter], vhl.wait.times.cens.F[,1,iter]), g.sf)
    alpha.1[iter] <- update.1[1]
    beta.1[iter] <- update.1[2]
    accepted.1[iter] <- update.1[3]

    update.3 <- gamma.parameter.GS(alpha.3[iter-1], beta.3[iter-1], 0,1,1,1, c(vhl.wait.times.cens.T[,2,iter], vhl.wait.times.cens.F[,2,iter]), g.sf)
    alpha.3[iter] <- update.3[1]
    beta.3[iter] <- update.3[2]
    accepted.3[iter] <- update.3[3]

    # Update rate of VHL driver mutations contributing to Z2
    # Taken from conjugate prior to the exponential (ie gamma) with uninformative priors
    lambda[iter] <- rgamma(1, shape = 0.01 + length(c(spor.wait.times.cens.F[,2,iter], spor.wait.times.cens.T[,2,iter])), rate = 0.01 + sum(c(spor.wait.times.cens.F[,2,iter], spor.wait.times.cens.T[,2,iter])))

    # Sample alternate waiting times for clones of different sizes
    sampled.wait.times.less0[,iter] <- sample.alternate.wait.times(spor.wait.times.cens.F[,,iter], spor.wait.times.cens.T[,,iter], lambda[iter], 1)
    sampled.wait.times.less0.25[,iter] <- sample.alternate.wait.times(spor.wait.times.cens.F[,,iter], spor.wait.times.cens.T[,,iter], lambda[iter], 0.75)
    sampled.wait.times.less0.5[,iter] <- sample.alternate.wait.times(spor.wait.times.cens.F[,,iter], spor.wait.times.cens.T[,,iter], lambda[iter], 0.5)
    sampled.wait.times.less0.75[,iter] <- sample.alternate.wait.times(spor.wait.times.cens.F[,,iter], spor.wait.times.cens.T[,,iter], lambda[iter], 0.25)

  }

  return(list(alpha.1, beta.1, alpha.3, beta.3, accepted.1, accepted.3, vhl.wait.times.cens.F, vhl.wait.times.cens.T, spor.wait.times.cens.T, spor.wait.times.cens.F, lambda, sampled.wait.times.less0, sampled.wait.times.less0.25, sampled.wait.times.less0.5, sampled.wait.times.less0.75))
}

######################################################################
censored.wait.times.GS <- function(curr.WT, d.sf, alphas, betas) {
  # Function to provide updates of estimated waiting times for censored cases (ie observed ages of incidence)
  # Done by Metropolis-Hastings with proposal distribution as Dirichlet centred on curr Z1, Z2 & Z3 and scaled
  # Choice of Dirichlet enforces constraint that waiting times must sum to the age of incidence (with scaling)
  # curr.WT is matrix of the current estimates of waiting times
  # d.sf is the scaling factor for the Dirichlet proposal distribution
  # alphas is a vector of the current alpha values for the gamma distributions of waiting times
  # betas is a vector of the current beta values for the gamma distributions of waiting times

  # Generate proposed ages
  proposal <- t(sapply(1:nrow(curr.WT), 
                            function(i,x) rdirichlet(1, d.sf * x[i,] / sum(x[i,])),
                            x=curr.WT))
  obs.ages <- rowSums(curr.WT)
  proposal.scaled <- proposal * obs.ages

  # Calculate importance ratios
  log.proposal.dist.ratio <- ddirichlet(curr.WT / obs.ages, d.sf * proposal, log=TRUE) - 
      ddirichlet(proposal, d.sf * curr.WT / obs.ages, log=TRUE)

  log.density.ratio <- rowSums( t((alphas-1) * t(log(proposal.scaled) - log(curr.WT)) - betas * t(proposal.scaled - curr.WT)) )

  importance.ratio <- exp(log.proposal.dist.ratio + log.density.ratio)

  if (!all(!is.na(importance.ratio))) {
    importance.ratio[is.na(importance.ratio)] <- 0
  }

  draws <- runif(n=nrow(curr.WT))
  curr.WT[draws < importance.ratio,] <- proposal.scaled[draws < importance.ratio,] 
  return(curr.WT)
}

######################################################################
gamma.parameter.GS <- function(curr.alpha, curr.beta, log.hyper.p, hyper.q, hyper.r, hyper.s, x, gamma.sf) {
  # Function to return updates on gamma parameters from its conjugate prior
  # curr.alpha and curr.beta are the current values of the alpha and beta parameters
  # log.hyper.p, hyper.q, hyper.r and hyper.s are hyperparameters for the conjugate prior for curr.alpha & curr.beta
  # x is a vector of the data values
  # gamma.sf is the scaling factor for the M-H algorithm

  # Up-date alpha & beta from the conjugate prior 
  # Due to the unnormalised nature of the conjugate, we sample using Metropolis-Hastings since the normalisation factor will cancel out
  # Use independent gamma proposal distributions for alpha and beta
  alpha.proposed <- rgamma(n=1, curr.alpha * gamma.sf, gamma.sf)
  beta.proposed <- rgamma(n=1, curr.beta * gamma.sf, gamma.sf)

  proposal.dist.ratio <- dgamma(x = curr.alpha, shape = alpha.proposed * gamma.sf, rate = gamma.sf) * 
    dgamma(x = curr.beta, shape = beta.proposed * gamma.sf, rate = gamma.sf) /
    dgamma(x = alpha.proposed, shape = curr.alpha * gamma.sf, rate = gamma.sf) /
    dgamma(x = beta.proposed, shape = curr.beta * gamma.sf, rate = gamma.sf)

  log.post.p <- log.hyper.p + sum(log(x))
  post.q <- hyper.q + sum(x)
  post.r <- hyper.r + length(x)
  post.s <- hyper.s + length(x)

  log.density.ratio <- (alpha.proposed - curr.alpha) * log.post.p - 
    post.q * (beta.proposed - curr.beta) - 
    post.r * (lgamma(alpha.proposed) - lgamma(curr.alpha)) +
    post.s * (alpha.proposed * log(beta.proposed) - curr.alpha * log(curr.beta))

  importance.ratio <- proposal.dist.ratio * exp(log.density.ratio)
  if (runif(n=1) < importance.ratio) {
    new.alpha <- alpha.proposed
    new.beta <- beta.proposed
    accepted <- TRUE
  } else {
    new.alpha <- curr.alpha
    new.beta <- curr.beta
    accepted <- FALSE
  }

  return(c(new.alpha, new.beta, accepted))
}

######################################################################
sample.alternate.wait.times <- function(WT.cens.F, WT.cens.T, lambda, lambda.fraction) {
  # Function to sample alternate wait times as if number of cells were lambda.fraction x current estimate
  WT.cens.F[,2] <- rexp(nrow(WT.cens.F), rate = lambda.fraction * lambda)
  WT.cens.T[,2] <- rexp(nrow(WT.cens.T), rate = lambda.fraction * lambda)
  return(rowSums(rbind(WT.cens.F, WT.cens.T)))
}

######################################################################
# Run the Gibbs sampler

age.inc.gs <- rcc.age.gs(spor.ages = sporadic.incidence, vhl.ages = vhl.incidence, chr3p.ages = t3_5.abs.time$triploid.est, d.sf=100, iterations=rcc.iters, g.sf = 10)
save(age.inc.gs, file = "age_inc_gs.RData")
rm(age.inc.gs)
```

### Checking convergence of MCMC chain

We check some convergence and mixing plots for the \(\alpha\) and \(\beta\) estimates. They show rather slow mixing for alpha\_1 and beta\_1. This is perhaps not surprising since the information for the \(Z\_1\) waiting time is somewhat confounded with the \(Z\_3\) waiting time, except through the prior information provided from the timings of t(3;-) translocations. Nonetheless, we have experimented with many different starting points for these values, and the posterior always converges well to the distribution shown here.

```
load("age_inc_gs.RData")

par(mfrow=c(2,2))
plot(1:rcc.iters, age.inc.gs[[1]], type="l", xlab="Iteration", ylab="Value drawn", main = "alpha_1")
plot(1:rcc.iters, age.inc.gs[[2]], type="l", xlab="Iteration", ylab="Value drawn", main = "beta_1")
plot(1:rcc.iters, age.inc.gs[[3]], type="l", xlab="Iteration", ylab="Value drawn", main = "alpha_3")
plot(1:rcc.iters, age.inc.gs[[4]], type="l", xlab="Iteration", ylab="Value drawn", main = "beta_3")
```

### Exploring outputs from Bayesian model

The key question of interest in this modelling are to establish the potential number of cells that carry chr 3p loss without having the other *VHL* allele mutated. The MCMC draws from the posterior distribution converge and mix very well as shown in the first plot below. As can be seen from the histogram in the second plot, the posterior distribution suggests that the number of cells in the kidney that carry chromosome 3p loss before the other *VHL* allele is mutated is only in the hundreds. That is, there is not a massive clonal expansion after the initiating event.

The other question of interest is to model what would happen to the age-incidence curves for sporadic kidney cancer if we had a treatment that could kill a fraction of cells at the chr 3p loss stage. This is shown in the bottom three plots below. It seems that we could halve the incidence cancer with a treatment in early adulthood that reduced the number of cells with chr 3p loss by 50%.

```
par(mfrow=c(1,1))
plot(1:rcc.iters, age.inc.gs[[11]] / vhl.driver.mutation.rate, type="l", xlab="Iteration", ylab="Value drawn", main = "Convergence plot of number of cells with 3p loss")
```

```
hist(age.inc.gs[[11]][rcc.burn.in:rcc.iters] / vhl.driver.mutation.rate, breaks=20, probability = TRUE, xlab = "Number of cells with 3p loss before VHL mutation", ylab="Density", main = "Posterior distribution of number of cells with 3p loss")
```

```
age.in.dens <- density(age.inc.gs[[11]][rcc.burn.in:rcc.iters] / vhl.driver.mutation.rate)
plot(age.in.dens, xlab = "Number of cells with 3p loss before VHL mutation", ylab="Density", main = "Posterior distribution of number of cells with 3p loss")
# polygon(age.in.dens, col="lightblue", border="black")
polygon(age.in.dens, col=adjustcolor("lightblue",alpha.f=0.2), border=adjustcolor("lightblue",alpha.f=0.8))
```

```
post.less0 <- apply(age.inc.gs[[12]][,(rcc.burn.in+1):rcc.iters], MARGIN = 2, FUN=sort)
post.less0 <- t(apply(post.less0, MARGIN = 1, FUN=quantile, probs=c(0.025,0.5,0.975)))
baseline.survfit <- survfit(Surv(post.less0[,2], rep(1, length(post.less0[,2]))) ~ 1)

post.less25 <- apply(age.inc.gs[[13]][,(rcc.burn.in+1):rcc.iters], MARGIN = 2, FUN=sort)
post.less25 <- t(apply(post.less25, MARGIN = 1, FUN=quantile, probs=c(0.025,0.5,0.975)))
less25.survfit.0.025 <- survfit(Surv(post.less25[,1], rep(1, length(post.less25[,1]))) ~ 1)
less25.survfit.0.5 <- survfit(Surv(post.less25[,2], rep(1, length(post.less25[,1]))) ~ 1)
less25.survfit.0.975 <- survfit(Surv(post.less25[,3], rep(1, length(post.less25[,1]))) ~ 1)
plot(NULL, xlim=c(0,90), ylim=c(0.95,1), xlab="Age (years)", ylab="Fraction free of kidney cancer", las=1, main="Decrease in clone size of 25%")
polygon(x=c(0,less25.survfit.0.025$time, rev(less25.survfit.0.975$time),0), y=c(1,less25.survfit.0.025$surv, rev(less25.survfit.0.975$surv),1), col="lightblue", border=NA)
lines(c(0,baseline.survfit$time), c(1,baseline.survfit$surv), lwd=3)
lines(c(0,less25.survfit.0.5$time), c(1,less25.survfit.0.5$surv), lwd=3, col="darkblue")
legend(10,0.97, lwd=c(3,3,0), col=c("black", "darkblue", NULL), fill=c(NA, NA, "lightblue"), border = "white", legend=c("Baseline incidence", "Incidence if clone size decreased by 25%", "95% posterior interval"), bty="n", merge=TRUE)
```

```
post.less50 <- apply(age.inc.gs[[14]][,(rcc.burn.in+1):rcc.iters], MARGIN = 2, FUN=sort)
post.less50 <- t(apply(post.less50, MARGIN = 1, FUN=quantile, probs=c(0.025,0.5,0.975)))
less50.survfit.0.025 <- survfit(Surv(post.less50[,1], rep(1, length(post.less50[,1]))) ~ 1)
less50.survfit.0.5 <- survfit(Surv(post.less50[,2], rep(1, length(post.less50[,1]))) ~ 1)
less50.survfit.0.975 <- survfit(Surv(post.less50[,3], rep(1, length(post.less50[,1]))) ~ 1)
plot(NULL, xlim=c(0,90), ylim=c(0.95,1), xlab="Age (years)", ylab="Fraction free of kidney cancer", las=1, main="Decrease in clone size of 50%")
polygon(x=c(0,less50.survfit.0.025$time, rev(less50.survfit.0.975$time),0), y=c(1,less50.survfit.0.025$surv, rev(less50.survfit.0.975$surv),1), col="lightblue", border=NA)
lines(c(0,baseline.survfit$time), c(1,baseline.survfit$surv), lwd=3)
lines(c(0,less50.survfit.0.5$time), c(1,less50.survfit.0.5$surv), lwd=3, col="darkblue")
legend(10,0.97, lwd=c(3,3,0), col=c("black", "darkblue", NULL), fill=c(NA, NA, "lightblue"), border = "white", legend=c("Baseline incidence", "Incidence if clone size decreased by 50%", "95% posterior interval"), bty="n", merge=TRUE)
```

```
post.less75 <- apply(age.inc.gs[[15]][,(rcc.burn.in+1):rcc.iters], MARGIN = 2, FUN=sort)
post.less75 <- t(apply(post.less75, MARGIN = 1, FUN=quantile, probs=c(0.025,0.5,0.975)))
less75.survfit.0.025 <- survfit(Surv(post.less75[,1], rep(1, length(post.less75[,1]))) ~ 1)
less75.survfit.0.5 <- survfit(Surv(post.less75[,2], rep(1, length(post.less75[,1]))) ~ 1)
less75.survfit.0.975 <- survfit(Surv(post.less75[,3], rep(1, length(post.less75[,1]))) ~ 1)
plot(NULL, xlim=c(0,90), ylim=c(0.95,1), xlab="Age (years)", ylab="Fraction free of kidney cancer", las=1, main="Decrease in clone size of 75%")
polygon(x=c(0,less75.survfit.0.025$time, rev(less75.survfit.0.975$time),0), y=c(1,less75.survfit.0.025$surv, rev(less75.survfit.0.975$surv),1), col="lightblue", border=NA)
lines(c(0,baseline.survfit$time), c(1,baseline.survfit$surv), lwd=3)
lines(c(0,less75.survfit.0.5$time), c(1,less75.survfit.0.5$surv), lwd=3, col="darkblue")
legend(10,0.97, lwd=c(3,3,0), col=c("black", "darkblue", NULL), fill=c(NA, NA, "lightblue"), border = "white", legend=c("Baseline incidence", "Incidence if clone size decreased by 75%", "95% posterior interval"), bty="n", merge=TRUE)
```

## Versioning notes

```
Sys.time()
```

```
## [1] "2017-07-17 14:13:39 BST"
```

```
sessionInfo()
```

```
## R version 3.4.0 (2017-04-21)
## Platform: x86_64-pc-linux-gnu (64-bit)
## Running under: Ubuntu 14.04.5 LTS
## 
## Matrix products: default
## BLAS: /usr/local/users/na11/local/lib/R/lib/libRblas.so
## LAPACK: /usr/local/users/na11/local/lib/R/lib/libRlapack.so
## 
## locale:
##  [1] LC_CTYPE=en_GB.UTF-8       LC_NUMERIC=C              
##  [3] LC_TIME=en_GB.UTF-8        LC_COLLATE=en_GB.UTF-8    
##  [5] LC_MONETARY=en_GB.UTF-8    LC_MESSAGES=en_GB.UTF-8   
##  [7] LC_PAPER=en_GB.UTF-8       LC_NAME=C                 
##  [9] LC_ADDRESS=C               LC_TELEPHONE=C            
## [11] LC_MEASUREMENT=en_GB.UTF-8 LC_IDENTIFICATION=C       
## 
## attached base packages:
## [1] stats     graphics  grDevices utils     datasets  methods   base     
## 
## other attached packages:
##  [1] abind_1.4-5      extraDistr_1.8.5 MCMCpack_1.4-0   coda_0.19-1     
##  [5] survival_2.41-3  pander_0.6.0     lme4_1.1-13      Matrix_1.2-10   
##  [9] MASS_7.3-47      reshape2_1.4.2   ggplot2_2.2.1    knitr_1.16      
## 
## loaded via a namespace (and not attached):
##  [1] Rcpp_0.12.11       magrittr_1.5       splines_3.4.0     
##  [4] munsell_0.4.3      colorspace_1.3-2   lattice_0.20-35   
##  [7] rlang_0.1.1        minqa_1.2.4        highr_0.6         
## [10] stringr_1.2.0      plyr_1.8.4         mcmc_0.9-5        
## [13] tools_3.4.0        grid_3.4.0         gtable_0.2.0      
## [16] nlme_3.1-131       quantreg_5.33      MatrixModels_0.4-1
## [19] lazyeval_0.2.0     digest_0.6.12      tibble_1.3.3      
## [22] nloptr_1.0.4       codetools_0.2-15   evaluate_0.10     
## [25] labeling_0.3       stringi_1.1.5      compiler_3.4.0    
## [28] scales_0.4.1       SparseM_1.77
```
