# Supplementary material for: Timing the Landmark Events in the Evolution of Clear Cell Renal Cell Cancer: TRACERx Renal
Source: Cell. 2018 Apr 19;173(3):611–623.e17. doi: 10.1016/j.cell.2018.02.020 (PMC5927631; doi:10.1016/j.cell.2018.02.020)
Supplement: Data S1. R Code for Timing Analysis, Related to Figure 5 — Interwoven R code, discussion and results for analysis of molecular and chronological timing of landmark events in ccRCC evolution. The document was created using the ‘knitr’ package. [file mmc7.zip › Supplemental Code/TCGA.mRNA.html]

TCGA mRNA analysis


# TCGA mRNA analysis

#### *Tom Mitchell*

#### *19 September 2017*

Load packages required

```
# source("http://bioconductor.org/biocLite.R")
# biocLite("TCGAbiolinks")
# biocLite("edgeR")
# biocLite("org.Hs.eg.db")
# biocLite("TxDb.Hsapiens.UCSC.hg19.knownGene")
# biocLite("ggbio")
library(TCGAbiolinks)
library(edgeR)
library(org.Hs.eg.db)
library(TxDb.Hsapiens.UCSC.hg19.knownGene)
library(ggbio)
```

## Introduction

This document investigates the relationship between gene expression and the presence/ mechanism of 5q gain. We rely on the publically available TCGA transciptional profiling, using the HTSeq format. The genomic features that we compare to results from the Battenberg (copy number) and Brass (structural variant) analyses of the matched TCGA whole genomes.

We aim to determine whether:  
1. We can detect increased transcription of genes that are present on the duplicated arm of chromosome 5q. Do any of the significantly differentially expressed genes match those thought to be putative targets in the literature?  
2. There are any significantly differentially expressed genes in the region of the unbalanced t(3;5) translocation.

## Load data

We load samples that we have already analysed WGS data (see main text) and have annotated according to t(3;5) status and whether there is gain of 5q (of any mechanism).

```
setwd("C:/Users/tjm/Google Drive/Current research/Renal/TCGA/RNA")
features <- read.delim("features.txt", header = T)
```

We can download the HTSeq count data directly from the TCGA using the TCGAbiolinks package from Bioconductor

```
query <- GDCquery(project = "TCGA-KIRC", 
                  data.category = "Transcriptome Profiling", 
                  data.type = "Gene Expression Quantification", 
                  workflow.type = "HTSeq - Counts",
                  barcode = features[,2])
GDCdownload(query)
KIRCseq <- GDCprepare(query)

KIRCMatrix <- TCGAanalyze_Preprocessing(KIRCseq)
colnames(KIRCMatrix) <- substr(colnames(KIRCMatrix),1,16)

write.table(KIRCMatrix, "C:/Users/tjm/Google Drive/Current research/Renal/TCGA/RNA/Outputs/TCGA_HTseq_Counts.genes.txt", sep="\t", quote=F, row.names=T)
```

## Processing mRNA data

For an initial filter, we remove all samples with fewer than a total of 20e6 reads. We also remove genes that do not have greater than 0.5 counts per million values in more than two samples. We convert to a EdgeR object for the subsequent analysis.

```
# remove samples with fewer than 2e7 counts
keep <- colnames(KIRCMatrix)[colSums(KIRCMatrix) > 2e7]
features <- features[features$id_2 %in% keep,]
KIRCMatrix <- KIRCMatrix[,colnames(KIRCMatrix) %in% keep]

KIRCCpm <- cpm(KIRCMatrix)


thresh <- KIRCCpm > 0.5
keep <- rowSums(thresh) >= 2
# Subset the rows of countdata to keep the more highly expressed genes
counts.keep <- KIRCMatrix[keep,]
## Convert to an edgeR object
dgeObj <- DGEList(counts.keep)
## Perform TMM normalisation
dgeObj <- calcNormFactors(dgeObj)
```

We now use genomic features of chromosome 5q status via a design matrix to annotate the principle component or MDS plot, in terms of biological coefficient of variance. This shows no gross correlation between the tranlocative 5q gains, the isolated 5q gains and diploid 5q status.

```
t3_5 <- features[,3]
gain_5 <- features[,7]

design <- model.matrix(~ t3_5 + gain_5)


plotMDS(dgeObj, labels=NULL, pch=rowSums(design), cex=0.75, xlim=c(-4, 5))
legend("topleft", pch=1:3, c("Diploid 5q", "Isolated 5q gain", "Translocative 5q gain"))
```

The common dispersion estimates the biological coefficient of variation of the dataset.

```
dgeObj <- estimateCommonDisp(dgeObj)
dgeObj <- estimateGLMTrendedDisp(dgeObj)
dgeObj <- estimateTagwiseDisp(dgeObj)
dgeObj <- estimateDisp(dgeObj, design = design)
### need to find the additional design term we used....
plotBCV(dgeObj)
```

## Testing of differential expression

We next calculate the likelihood ratios for differential expression of individual genes, comparing samples with chromosome 5 gains with those with diploid chromosome 5.

```
fit <- glmFit(dgeObj, design)

####  coef=3 compares gain of chromosome 5 vs diploid, coef=2 compares gain of chromosome 5 via t(3;5) vs non-t(3;5)
coef = 3
lrt.gvsW <- glmLRT(fit, coef=coef)
# topTags(lrt.gvsW)
```

We visulise the results of the differential expression analysis, plotting the log-fold change against log-counts per million.

```
## The total number of genes - down-regulated, unchanged and up-regulated
summary(de <- decideTestsDGE(lrt.gvsW))
```

```
##    gain_5TRUE
## -1        122
## 0       22586
## 1         125
```

```
detags <- rownames(dgeObj)[as.logical(de)]

### Annotate most differentially expressed genes
results_g <- as.data.frame(topTags(lrt.gvsW,n = Inf))
annot_g <- select(org.Hs.eg.db,keytype="ENSEMBL",keys=rownames(results_g), columns=c("ENSEMBL","SYMBOL","GENENAME", "ENTREZID"), multiVals="first")
annot_g <- annot_g[!duplicated(annot_g[,1]),]
results.annotated_g <- cbind(results_g, annot_g)

de <- decideTestsDGE(lrt.gvsW)
detags <- rownames(dgeObj)[as.logical(de)]
topTags <- results.annotated_g[rownames(de)[de[,1] !=0],]
plotSmear(lrt.gvsW, de.tags=detags, main="DE for samples with 5q gains compared to wildtype")
```

```
#text(topTags$logCPM, topTags$logFC, topTags$SYMBOL, col="blue", cex=0.8)
```

We next convert our annotated gene based results to genomic region. We then plot the differential expression for all genes across the entire genome. The karyogram hightlights the unsurprising inbalance with preferential upregulation of genes across the entirety of chromosome 5.

```
tx <- TxDb.Hsapiens.UCSC.hg19.knownGene
exo <- exonsBy(tx, "gene")
sigGenes <- results.annotated_g[detags,]
exoRanges <- unlist(range(exo))
sigRegions <- exoRanges[na.omit(match(sigGenes$ENTREZID, names(exoRanges)))]
allRegions <- exoRanges[na.omit(match(results.annotated_g$ENTREZID, names(exoRanges)))]
mcols(sigRegions) <- sigGenes[match(names(sigRegions), sigGenes$ENTREZID),]
mcols(allRegions) <- results.annotated_g[match(names(allRegions), results.annotated_g$ENTREZID),]

sigRegions <- keepSeqlevels(sigRegions, paste0("chr", c(1:22,"X","Y")))
allRegions <- keepSeqlevels(allRegions, paste0("chr", c(1:22,"X","Y")), pruning.mode = "coarse")
sigRegions <- sigRegions[order(sigRegions$LR,decreasing = TRUE)]
## We can visualise the significantly differentially expressed genes
#plotGrandLinear(sigRegions , aes(y = logFC))
## We can visualise all expressed genes
plotGrandLinear(allRegions , aes(y = logFC))
```

```
## or as a karyogram...
mcols(sigRegions)$UpRegulated <- mcols(sigRegions)$logFC > 0
autoplot(sigRegions,layout="karyogram",aes(color=UpRegulated,
                                       fill=UpRegulated))
```

To compare the number of differentially expressed genes between chromosomes in a more simple manner, we first normalise the absolute number according to the number of quantified genes per chromosome, before plotting as a barplot.

```
### Generation of a summary table for the number of down-regulated versus downregulated genes per chromosome
status <- table(as.data.frame(sigRegions)$seqnames, as.data.frame(sigRegions)$UpRegulated)
### Number of genes per chromosome to be used to normalise the number of dysregulated genes
no.genes <- table(as.data.frame(allRegions, row.names=NULL)$seqnames)
status[,1] <- -status[,1]
### Barplot to show summary by chromosome
barplot(t(status/ as.vector(no.genes))[1,]*mean(no.genes), beside=T, ylim=c(-10, 30), col="red",
        names.arg=c(1:22, "X", "Y"), legend.text = c("Up-regulated", "Down-regulated"),
        args.legend = list(fill=c("blue", "red")), xlab="Chromosome",
        ylab="Normalised differentially expressed genes, n")
barplot(t(status/ as.vector(no.genes))[2,]*mean(no.genes), names="", beside=T, add=T, col="blue")
```

```
chr5 <- as.data.frame(sigRegions)[as.data.frame(sigRegions)$seqnames %in% "chr5",]
write.table(chr5, "C:/Users/tjm/Google Drive/Current research/Renal/TCGA/RNA/Outputs/DE_chr5.genes.txt", sep="\t", quote=F, row.names=F)
results.annotated_g[results.annotated_g$SYMBOL %in% "SQSTM1",]
```

```
##                     logFC   logCPM        LR    PValue       FDR
## ENSG00000161011 0.1190076 8.396617 0.4066863 0.5236562 0.8776215
##                         ENSEMBL SYMBOL       GENENAME ENTREZID
## ENSG00000161011 ENSG00000161011 SQSTM1 sequestosome 1     8878
```

We now plot the location of DE genes in chromosome 5, to determine whether there is clustering close to the t(3;5) breakpoints (80-129 Mb).

```
autoplot(sigRegions,layout="karyogram", chr="chr5",
         aes(color=UpRegulated, fill=UpRegulated))
```

We have shown that, perhaps unsurprisingly, chromosomal 5 gains result in a general upregulation of genes on chromosome 5. There is no evidence of clustering of differentially expressed genes close to the t(3;5) breakpoints. We now investigate whether the t(3;5) translocations result in a different transcriptional landscape to non-t(3;5) tumours by repeating the above analysis comparing t(3;5) tumours with non-t(3;5) tumours (this will include these with and without whole chromosomal 5 gains).

```
fit <- glmFit(dgeObj, design)

####  coef=3 compares gain of chromosome 5 vs diploid, coef=2 compares gain of chromosome 5 via t(3;5) vs non-t(3;5)
coef = 2
lrt.gvsW <- glmLRT(fit, coef=coef)

## The total number of genes - down-regulated, unchanged and up-regulated
summary(de <- decideTestsDGE(lrt.gvsW))
```

```
##    t3_5TRUE
## -1       44
## 0     22764
## 1        25
```

```
detags <- rownames(dgeObj)[as.logical(de)]

### Annotate most differentially expressed genes
results_g <- as.data.frame(topTags(lrt.gvsW,n = Inf))
annot_g <- select(org.Hs.eg.db,keytype="ENSEMBL",keys=rownames(results_g), columns=c("ENSEMBL","SYMBOL","GENENAME", "ENTREZID"), multiVals="first")
annot_g <- annot_g[!duplicated(annot_g[,1]),]
results.annotated_g <- cbind(results_g, annot_g)

de <- decideTestsDGE(lrt.gvsW)
detags <- rownames(dgeObj)[as.logical(de)]
topTags <- results.annotated_g[rownames(de)[de[,1] !=0],]
plotSmear(lrt.gvsW, de.tags=detags, main="DE for samples with 5q gains compared to wildtype")
```

```
#text(topTags$logCPM, topTags$logFC, topTags$SYMBOL, col="blue", cex=0.8)

tx <- TxDb.Hsapiens.UCSC.hg19.knownGene
exo <- exonsBy(tx, "gene")
sigGenes <- results.annotated_g[detags,]
exoRanges <- unlist(range(exo))
sigRegions <- exoRanges[na.omit(match(sigGenes$ENTREZID, names(exoRanges)))]
allRegions <- exoRanges[na.omit(match(results.annotated_g$ENTREZID, names(exoRanges)))]
mcols(sigRegions) <- sigGenes[match(names(sigRegions), sigGenes$ENTREZID),]
mcols(allRegions) <- results.annotated_g[match(names(allRegions), results.annotated_g$ENTREZID),]

sigRegions <- keepSeqlevels(sigRegions, paste0("chr", c(1:22,"X","Y")))
allRegions <- keepSeqlevels(allRegions, paste0("chr", c(1:22,"X","Y")), pruning.mode = "coarse")
sigRegions <- sigRegions[order(sigRegions$LR,decreasing = TRUE)]
## We can visualise the significantly differentially expressed genes
#plotGrandLinear(sigRegions , aes(y = logFC))
## We can visualise all expressed genes
#plotGrandLinear(allRegions , aes(y = logFC))
## or as a karyogram...
mcols(sigRegions)$UpRegulated <- mcols(sigRegions)$logFC > 0
autoplot(sigRegions,layout="karyogram",aes(color=UpRegulated,
                                       fill=UpRegulated))
```

```
### Generation of a summary table for the number of down-regulated versus downregulated genes per chromosome
status <- table(as.data.frame(sigRegions)$seqnames, as.data.frame(sigRegions)$UpRegulated)
### Number of genes per chromosome to be used to normalise the number of dysregulated genes
no.genes <- table(as.data.frame(allRegions, row.names=NULL)$seqnames)
status[,1] <- -status[,1]
### Barplot to show summary by chromosome
barplot(t(status/ as.vector(no.genes))[1,]*mean(no.genes), beside=T, ylim=c(-10, 30), col="red",
        names.arg=c(1:22, "X", "Y"), legend.text = c("Up-regulated", "Down-regulated"),
        args.legend = list(fill=c("blue", "red")), xlab="Chromosome",
        ylab="Differentially expressed genes, (n)")
barplot(t(status/ as.vector(no.genes))[2,]*mean(no.genes), names="", beside=T, add=T, col="blue")
```
